# High efficacy of the F-ATP synthase inhibitor TBAJ-5307 against nontuberculous mycobacteria *in vitro* and *in vivo*

**DOI:** 10.1016/j.jbc.2023.105618

**Published:** 2024-01-03

**Authors:** Priya Ragunathan, Patcharaporn Sae-Lao, Claire Hamela, Matthéo Alcaraz, Alexander Krah, Wee Han Poh, Carmen Jia Ern Pee, Albert Yick Hou Lim, Scott A. Rice, Kevin Pethe, Peter J. Bond, Thomas Dick, Laurent Kremer, Roderick W. Bates, Gerhard Grüber

**Affiliations:** 1 School of Biological Sciences, Nanyang Technological University, Singapore; 2 School of Chemistry, Chemical Engineering and Biotechnology, Nanyang Technological University, Singapore; 3 Centre National de la Recherche Scientifique UMR 9004, Institut de Recherche en Infectiologie de Montpellier (IRIM), Université de Montpellier, Montpellier, France; 4 Bioinformatics Institute, Agency for Science, Technology and Research (A∗STAR), Singapore; 5 Singapore Centre for Environmental Life Sciences Engineering, Nanyang Technological University, Singapore; 6 Lee Kong Chian School of Medicine, Nanyang Technological University, Singapore; 7 Department for Respiratory and Critical Care Medicine, Tan Tock Seng Hospital, Singapore; 8 Microbiomes for One Systems Health and Agriculture and Food, CSIRO, Westmead, New South Wales, Australia; 9 National Centre for Infectious Diseases (NCID), Singapore; 10 Center for Discovery and Innovation, Hackensack Meridian Health, Nutley, New Jersey, USA; 11 Department of Medical Sciences, Hackensack Meridian School of Medicine, Nutley, New Jersey, USA; 12 Department of Microbiology and Immunology, Georgetown University, Washington, District of Columbia, USA; 13 INSERM, IRIM, Montpellier, France

**Keywords:** mycobacteria, nontuberculosis mycobacterium, antibiotics, bacterial pathogenesis, membrane protein, ATP synthesis

## Abstract

The F_1_F_O_-ATP synthase engine is essential for viability and growth of nontuberculous mycobacteria (NTM) by providing the biological energy ATP and keeping ATP homeostasis under hypoxic stress conditions. Here, we report the discovery of the diarylquinoline TBAJ-5307 as a broad spectrum anti-NTM inhibitor, targeting the F_O_ domain of the engine and preventing rotation and proton translocation. TBAJ-5307 is active at low nanomolar concentrations against fast- and slow-growing NTM as well as clinical isolates by depleting intrabacterial ATP. As demonstrated for the fast grower *Mycobacterium abscessus*, the compound is potent *in vitro* and *in vivo*, without inducing toxicity. Combining TBAJ-5307 with anti-NTM antibiotics or the oral tebipenem–avibactam pair showed attractive potentiation. Furthermore, the TBAJ-5307–tebipenem–avibactam cocktail kills the pathogen, suggesting a novel oral combination for the treatment of NTM lung infections.

Nontuberculous mycobacteria (NTM) most commonly cause pulmonary infections (90%), especially among patients with structural airway diseases like cystic fibrosis and bronchiectasis but can also cause lymphadenitis, skin and soft tissue infection, cardiac infection, bone and joint infections, and disseminated disease (1). Classically, NTM have been divided into rapid and slow growers. Notable rapid growers include *Mycobacterium abscessus* (*Mab*) complex (*Mab* subsp. *abscessus*, *Mab* subsp. *bolletii*, and *Mab* subsp. *massiliense*), *Mycobacterium fortuitum*, and *Mycobacterium mucogenicum*. Examples of slow growers causing human disease include the *Mycobacterium avium complex* (*e.g.*, *M. avium* and *M. intracellulare*), *Mycobacterium kansasii*, and *Mycobacterium xenopi* (2).

NTM infections are increasing exponentially in their global prevalence, morbidity, and mortality. Cell wall impermeability, along with biofilm formation, contributes to resistance against antimicrobials, high temperatures, and disinfectants. NTMs like *Mab* can exhibit either a smooth (S) or rough (R) morphotype as a consequence of the presence or the absence, respectively, of bacterial surface glycopeptidolipids (3). Importantly, S–R morphotypes show differences in virulence (2, 3). These morphological differences are also associated with important physiological differences. In addition, NTM harbor intrinsic resistance mechanisms, quickly acquire resistance, and display drug tolerance, which renders most antimicrobial classes ineffective. Cure rates are globally poor, comparable to or worse than those of multidrug-resistant tuberculosis (4).

Importantly, most NTM are able to enter a metabolically quiescent state for instance under low oxygen tension (hypoxia), where the pathogens phenotypically become more tolerant to drug challenge with existing drugs (4). Being obligate aerobes, NTM need to regulate under such hypoxia conditions the essential metabolic processes of oxidation and recycling of the electron carriers (NADH_2_ and FADH_2_), the generation of a proton motive force, and the formation of sufficient amounts of ATP. The latter is driven by the oxidative phosphorylation pathway, including the F_1_F_O_-ATP synthase catalyzing ATP formation (5). Since the ATP pool is reduced in such persister cells, they become exquisitely sensitive to any further ATP depletion and susceptible to drugs targeting the maintenance of an optimal ATP:ADP ratio. This observation suggests that inhibitors targeting the essential NTM F_1_F_O_-ATP synthase (α_3_:β_3_:γ:ε:*b-δ*:*b’*:*a*:*c*_*9*_; Fig. 1*A*) could overcome the problem of NTM drug tolerance and shortening the duration of therapy for drug-resistant NTM diseases. This working model is supported by the antituberculosis therapeutic bedaquiline (BDQ), consisting of a quinoline-, a phenyl-, a naphthyl ring, and a dimethyl amino tether (Fig. 1*B*), and targeting the *Mab* F-ATP synthase (6). While BDQ is used as salvage therapy against *Mab* lung diseases (7), it has been overshadowed by its pharmacokinetic properties and tolerability profiles as well as its inhibition of the mitochondrial F-ATP synthase in human embryonic kidney 293S cell mitoplasts (8). Recently, BDQ’s 3,5-dialkoxypyridine analog TBAJ-876 with improved pharmacokinetic and tolerability profiles has shown to be active *in vitro* and *in vivo* against the *Mab* complex (9).

**Figure 1:**
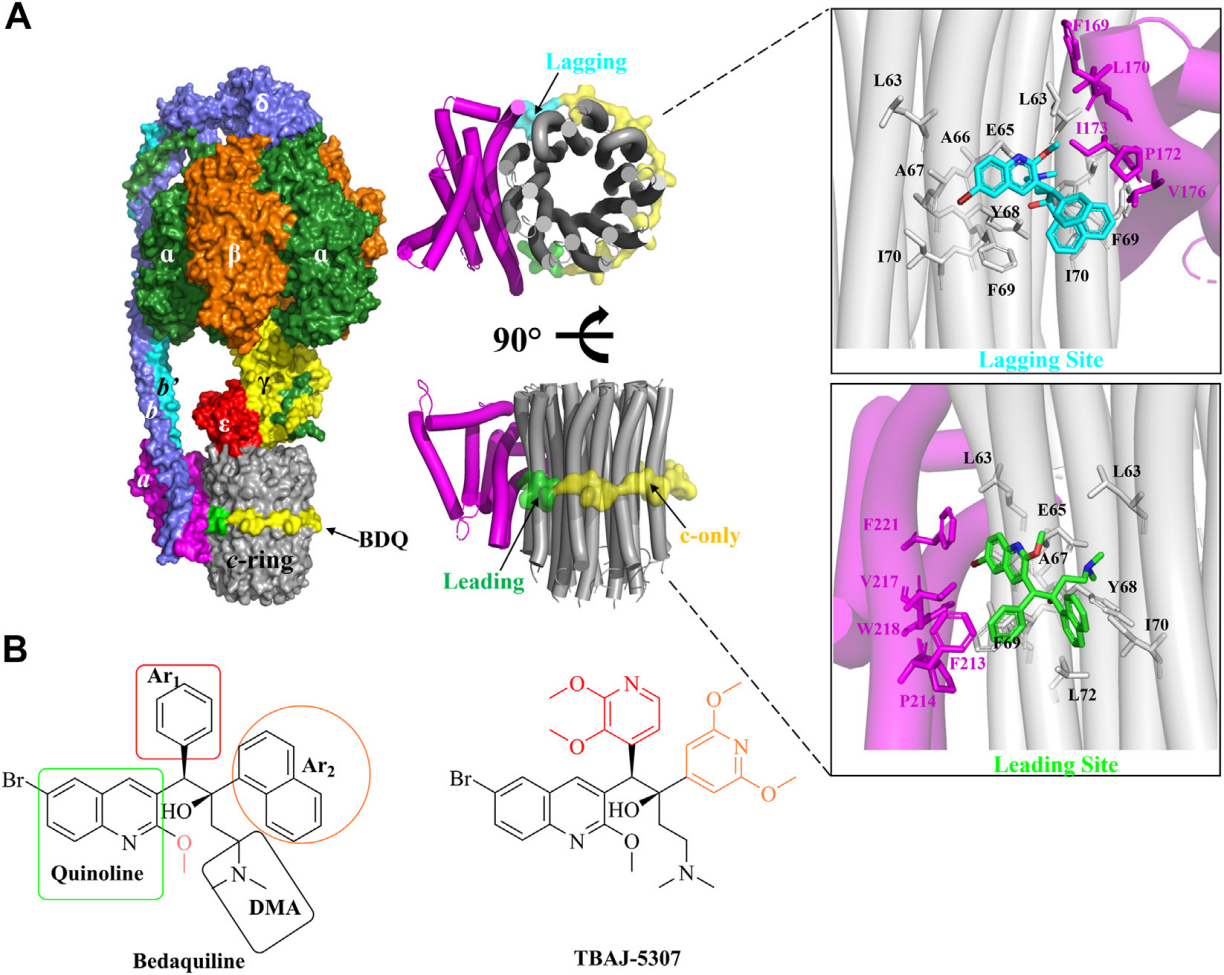
**Binding-sites of BDQ in the mycobacterial engine and the key chemical groups of BDQ and TBAJ-5307.***A*, *Mycobacterium smegmatis* F-ATP synthase cryo-EM structure with bound BDQ (Protein Data Bank ID: 7JGA). The hexamer forming α and β subunits is shown in *green* and *orange color*, respectively. The γ (*yellow*), ε (*red*), *b*δ (*blue*), *b’* (*cyan*), and *a* (*magenta*) subunits and *c*-ring (*gray*) are shown in surface representation. The seven BDQ molecules that bind in *c*-ring are also shown in surface representation. BDQ binds five *c*-only sites (*yellow*), a leading site (*green*), and a lagging site (*cyan*). For clarity, the *c*-ring along with subunit *a* are shown in *cartoon representation* in two views. In the zoomed-in view, the residues, which are interacting with BDQ in the leading site and lagging site, are shown. *B*, BDQ with its key pharmacophore features (a quinoline, a phenyl (Ar_1_), a naphthyl (Ar_2_) ring, and a dimethyl amino [DMA] tether). In comparison, TBAJ-5307 has a 2,3-dimethoxy-pyridin-4-yl moiety as Ar_1_ group and a 2,6-dimethoxy-pyridin-4-yl moiety as Ar_2_ group. BDQ, bedaquiline.

However, in contrast to *Mycobacterium tuberculosis*, both inhibitors are not bactericidal and have 100 times lower growth-inhibitory potency in *Mab*, proposed to be correlated in part with crossresistance in *Mab* efflux pumps (6). In addition, the cryo-EM structure of *Mycobacterium smegmatis* F_1_F_O_-ATP synthase, a surrogate of the *M. tuberculosis* enzyme, showed BDQ binding to three sites of the F_O_ domain, namely the leading sites and lagging sites and the *c*-ring, which binds five BDQ molecules (Fig. 1*A*) (10). The leading site involves a *c* subunit that has interacted with subunit *a* and picked up a proton from the periplasm, whereas the lagging site involves a *c* subunit poised to interact with subunit *a* to deposit a proton into the cytoplasm (10). The subunit *a* amino acid sequences of the three *Mab* subspecies differ from that of *M. smegmatis*, including the residues at the leading sites and lagging sites (Fig. S1).

Furthermore, the BDQ analogs TBAJ-5307, TBAJ-5316, and TBAJ-5366 with improved potency and physiochemical properties have been described (11). We showed that the racemates of (±)-5307, (±)-5316, and (±)-5366 (Cambridge Crystallographic Data Centre deposition numbers: 1967685, 1967683, 1967684, respectively; Fig. S2) exhibited improved growth and ATP synthesis inhibition against *M. smegmatis*, when compared with BDQ (12). Here, we demonstrate anti-*Mab* activity of the racemates of (±)-5307, (±)-5316, and (±)-5366 with (±)-5307 being the most potent one. Its enantiomer TBAJ-5307 displays high nanomolar efficacy against representatives of the fast and slow NTM growers, making it an attractive broad spectrum anti-NTM inhibitor. Its potency in cell growth and ATP synthesis inhibition is significantly higher compared with BDQ and TBAJ-876. Its mechanism of action is addressed by molecular dynamics (MD) simulations and binding free-energy calculations. TBAJ-5307 is efficacious *ex vivo* and *in vivo* without altering biofilm formation or being toxic and potentiates the activity of the clinical *Mab* antibiotics clofazimine (CFZ), amikacin, rifabutin (RFB), and the oral pair tebipenem (TBP) and avibactam (AVI). These features make TBAJ-5307 an ideal candidate to tackle the issue of NTM drug tolerance, high nanomolar anti-NTM potency, toxicity, pronounced combinatory efficacy with anti-*Mab* antibiotics to overcome intrinsic and acquired resistance as well as to design an oral cocktail in future, which reduces the expense and risks of intravenous therapies of today’s anti-NTM drugs.

## Results and discussion

### Anti-*Mab* activity of racemates (±)-5307, (±)-5316, and (±)-5366

**Figure 2:**
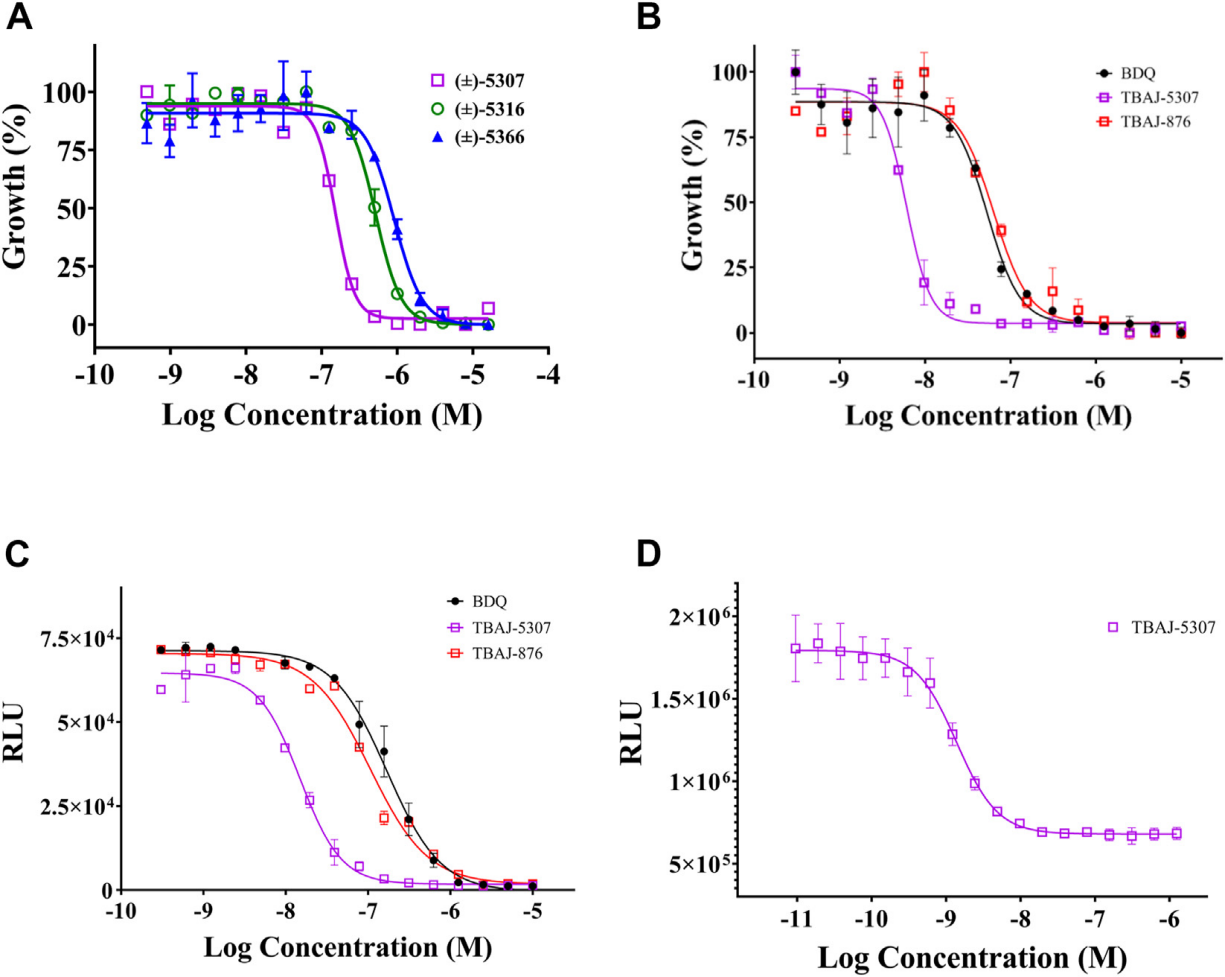
**Anti-*Mab* testing of BDQ analogs and racemates.***A*, growth inhibition dose–response curve of *Mab* subsp. *abscessus* ATCC 19977 cells by the racemates (±)-5307, (±)-5316, and (±)-5366. Two biological replicates were carried out each in three technical replicates. Data represent the average of all the experiments. Bacterial growth is expressed as percentage of control/untreated sample. *B*, growth inhibition dose–response curve of smooth morphotype strains of *Mab* subsp. *abscessus* ATCC 19977 by BDQ, TBAJ-876, and TBAJ-5307. Two biological replicates were carried out each in three technical replicates. Data represent the average of all the experiments. Bacterial growth is expressed as percentage of control/untreated sample. *C* and *D*, intracellular ATP synthesis inhibition of smooth (*C*) morphotype strains of *Mab* and (*D*) *Mab* IMVs. The ATP content of the cells was measured by adding BacTiter-Glo (Promega) to the cells. The total ATP content is directly proportional to relative luminescence units (RLUs). Two biological replicates were carried out each in three technical replicates. Data represent the average of all the experiments. ∗∗∗∗*p* < 0.0001, statistical analysis was carried out using two-way ANOVA test for all the experiments presented. ATCC, American Type Culture Collection; IMV, inverted-membrane vesicle; *Mab*, *Mycobacterium abscessus*.

Most of the administered anti-NTM drugs possess minimum inhibitory concentrations (MIC_50_) for growth inhibition in the micromolar range. To extend the poorly populated anti-NTM drug pipeline with a nanomolar potency inhibitor and the energy converter F-ATP synthase of the fast growing NTM *Mab* as a target, we first used a repurposing approach of our TBAJ-racemates (±)-5307, (±)-5316, and (±)-5366, which were synthesized according to Sutherland *et al.* (11) and Hotra *et al.* (12), and evaluated the susceptibility of *Mab* subsp. *abscessus* ATCC 19977 to the compounds in complete Middlebrook 7H9 broth. As shown in Figure 2*A*, the three racemates (±)-5307, (±)-5316, and (±)-5366 showed good growth inhibition with MIC_50_ values of 150 ± 40 nM, 510 ± 100 nM, and 900 ± 20 nM, respectively.

### High potency of TBAJ-5307 against reference and clinical strains of the *M. abscessus* complex

Since (±)-5307 was most potent among the three racemates, the enantiomers were resolved (see the Experimental procedures section). The levorotatory enantiomer, (−)-TBAJ5307, called TBAJ-5307, displayed high potency with MIC_50_ of 4.5 ± 0.9 nM and 6 ± 1.2 nM against the *M*. subsp. *abscessus* smooth strain (S variant; Fig. 2*B*) and rough strain (R variant) (Fig. S3*A*), respectively. The reasons for the high activity of this levorotatory compared with the racemate are currently under investigation. In contrast, the dextrorotatory enantiomer, (+)-TBAJ5307, showed no significant activity (Fig. S3*B*). Importantly, TBAJ-5307 showed a 12 times higher potency compared with BDQ (53 ± 8.2 nM and 74 ± 4 nM) or TBAJ-876 (22 ± 2.9 nM and 63 ± 3.8 nM). Since a potent anti-*Mab* agent requires potency against clinical isolates, the efficacy of TBAJ-5307 was also demonstrated against the isolate *Mab* Bamboo (13) (MIC_50_ = 16 ± 1.3 nM; Fig. S3*C* and Table 1). TBAJ-5307 also displayed high efficacy against *M. bolletii* and *M. massiliense* with MIC_50_ of 2.7 ± 0.9 nM and 5.2 ± 1.5 nM, respectively, underscoring that the compound is active against representative strains of the entire *Mab* complex (Fig. S3*D*). In addition, TBAJ-5307 inhibited growth of *M*. subsp. *massiliense* clinical isolate 2 and *M*. subsp. *massiliense* clinical isolate 1 with an MIC_50_ of 21.4 ± 1.8 nM and 36.1 ± 4.2 nM, respectively (Fig. S3*E*).

**Table 1:** Growth-inhibitory and ATP synthesis potency of BDQ, TBAJ-876, and TBAJ-5307 against NTM strains in 7H9 broth medium

NTM strain	MIC_50_ (nM)			IC_50_ (nM)		
	BDQ	TBAJ-876	TBAJ-5307	BDQ	TBAJ-876	TBAJ-5307
*Mab abscessus* (S)	53	22	4.5	60	106	7
*Mab abscessus* (R)	74	63	6.0	148	193	15
*Mab.* Bamboo	108	143	39	351	496	100
*Mab bolletii*	119	187	58	70	73	11
*Mab massilience*	141	246	38	230	467	73
*M. avium*	184	64	1.8	267	210	12
*M. avium isolate*	76	29	31	26	7	4.6
*M. intracellulare*	60	30	16	55	16	6.7
*M. fortuitum*	8.3	1.7	0.9	n.d.	n.d.	n.d.

The experiments were carried out three times independently.

Abbreviation: n.d., not determined.

To determine whether TBAJ-5307 is bactericidal against *Mab*, we measured the survival of the strain upon drug exposure. TBAJ-5307 was bacteriostatic against *M*. subsp. *abscessus* at 30-fold its MIC_50_ in 7H9 medium as BDQ (Fig. S3*F*) and as described for TBAJ-876 (9).

To determine whether *Mab* growth inhibition by TBAJ-5307 is related to depletion of ATP, intracellular ATP synthesis was measured. As shown in Figure 2*C* and Fig. S3*G*, TBAJ-5307 depleted ATP synthesis within the bacilli with an half-maximal inhibitory concentration (IC_50_) of 7 ± 0.3 nM and 15 ± 1.4 nM in the S and R variants of *M*. subsp. *abscessus*, respectively. In comparison, lower ATP synthesis inhibition of the S and R variants was observed in the presence of BDQ (60 ± 12.5 nM and 148 ± 9.6 nM) or TBAJ-876 (106 ± 20 nM and 193 ± 35 nM). These results underline that the improved growth inhibition by TBAJ-5307 is consistent with higher intracellular ATP depletion when compared with BDQ or TBAJ-876. Since oxygen consumption within the respiratory chain of *M*. subsp. *abscessus* is unaffected at 125 nM and 250 nM of TBAJ-5307 (Fig. S3*H*), the data also indicate that the inhibitor targets the *Mab* F-ATP synthase.

### TBAJ-5307’s target specificity and mechanism of action

To confirm that the observed whole-cell ATP depletion correlates with the inhibition of the *Mab* F_1_F_O_-ATP synthase only, ATP formation of inverted-membrane vesicles of *M*. subsp. *abscessus* R variant was measured, resulting in an IC_50_ of 1.4 ± 0.5 nM (Fig. 2*D*), which is comparable to the IC_50_ observed for the whole cell assay described previously (7 ± 0.3 nM; Table 1). This indicates that TBAJ-5307’s high potency of *Mab* growth inhibition correlates with the inhibition of *Mab’s* F-ATP synthase.

**Figure 3:**
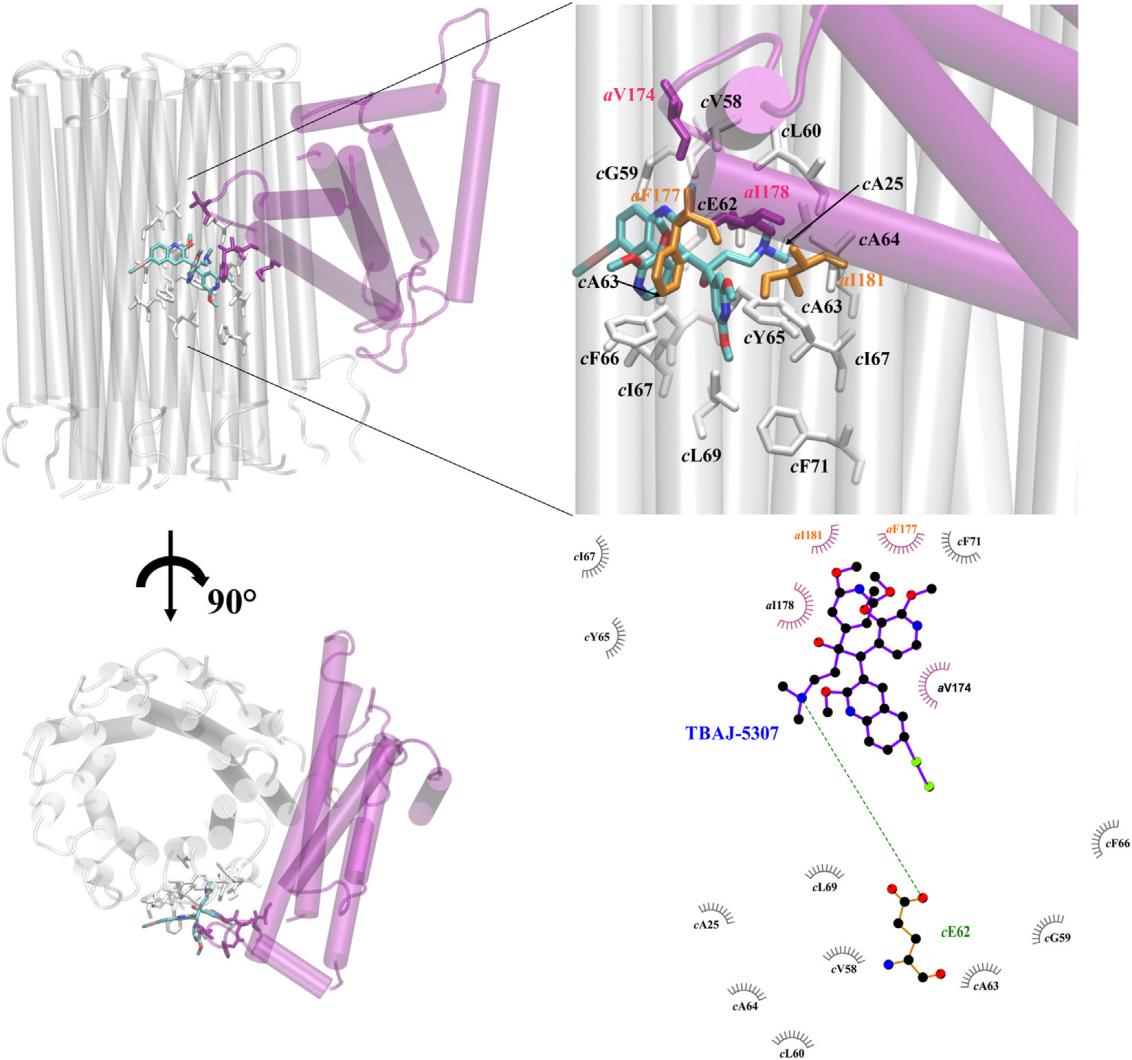
**Predicted binding of TBAJ-5307 to the *Mab* F**_**O**_**-lagging site.** Position of TBAJ-5307 bound to the F_O_ domain in the side view (*top left*) and top view (*bottom left*), respectively. Zoom into the binding site (*top right*) and the LigPlot+ representation (27) (*bottom right*) are shown. Residues of the *c*-ring are displayed in *gray*, and residues of subunit *a* are shown in *purple* (same residues as in *Mycobacterium smegmatis*) and *orange* (different residues than in *M. smegmatis*). Figures representing molecular representations were produced with VMD (visual molecular dynamics) (28). *Mab*, *Mycobacterium abscessus*.

To explore the inhibitory potency at an energetic level and to understand the mechanism of action, we performed MD simulations and free-energy calculations as successfully done previously for BDQ and TBAJ-876 binding to the *M. smegmatis* (14) and BDQ binding to the *Mab* F_O_ domains (15). First, the structure of the *Mab* F_O_ domain was modeled based on the cryo-EM structure of the *M. smegmatis* F_O_ part (10), and TBAJ-5307 was fitted on to BDQ, which was resolved in the *M. smegmatis* structure (Protein Data Bank ID: 7JGC) (10). While the *c*-ring residues are conserved between *M. smegmatis* and the three *Mab* subspecies, differences in subunit *a* at the leading and lagging sites can be observed (Fig. S1), which in part may explain the 100 times lower potency of BDQ and TBAJ-876 against *Mab* when compared with *M. smegmatis* (6, 9). The MD simulations (see supporting information: Computational procedures and data) revealed the binding site of TBAJ-5307 with emphasis on the lagging and leading sites, since they contribute mainly to the proposed inhibitory mechanism when compared with the *c*-ring-only site (10). TBAJ-5307 interacts with the *Mab c*-ring residues *c*A25, *c*V58, *c*G59, *c*L60, *c*E62, *c*A63, *c*A64, *c*Y65, *c*F66, *c*I67, and *c*L69 and subunit *a* residues *a*V174, *a*F177, *a*I178, and *a*I181 for the lagging site (Fig. 3), whereas in the leading site, residues *c*A25, *c*V58, *c*G59, *c*L60, *c*E62, *c*A63, *c*A64, *c*Y65, *c*F66, *c*I67, and *c*L69 and residues *a*A218, *a*P219, *a*I222, *a*W223, and *a*F226 interact with the inhibitor (Fig. S4 and Table S1). Interestingly, the *Mab*-specific residues *a*F177 and *a*I181 of the lagging site (*a*P172 and *a*V176 in *M. smegmatis*) form hydrophobic interactions with the 2,3-dimethoxy-pyridin-4-yl moiety of TBAJ-5307 (Fig. 3) and contribute to the strong binding of the agent, which hinders rotation of the *c*-ring turbine and movement of the proton to residue *a*R193 for further translocation *via* the second half-channel to the cytoplasmic side, a requirement for ATP formation. In comparison, the 2,6-dimethoxy-pyridin-4-yl moiety exhibits hydrophobic contacts with the *Mab*-specific amino acids *a*A218 and *a*I222 (*a*F213 and *a*V217 in *M. smegmatis*) in the leading site (Fig. S4).

Free energy calculations resulted in a binding free energy of −9.4 ± 1.3 kcal/mol compared with −12.0 kcal/mol derived from experimental data (Fig. 2*D*) for the lagging site; the offset is similar to that derived from our previous work for strong BDQ binding to *M. smegmatis* (14). The free-energy calculations are thus in reasonable agreement with the experimental data, thereby confirming the accuracy of the modeled complex. The offset in free energy likely results from the modeled neutral state of the compound and the key *c*E62; the alternative charged form would lead to artifactual membrane solvation, as discussed previously (16). For the leading site, we obtained a binding free energy of −9.8 ± 3.8 kcal/mol and thus predict a comparable inhibition of the rotation for the leading site.

**Figure 4:**
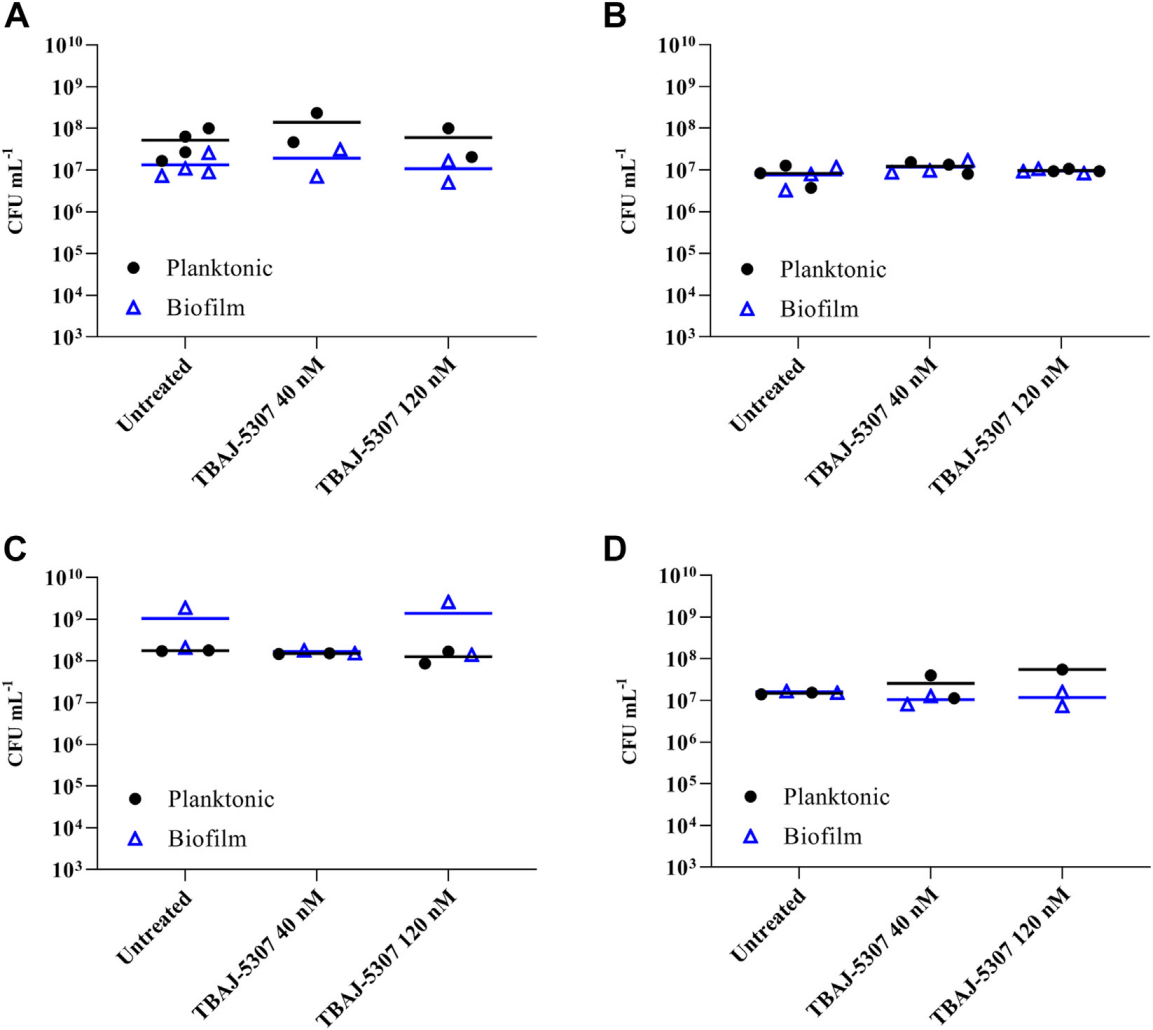
**Activity of 40 or 120 nM of TBAJ-5307 against 3 h preformed biofilm of *Pseudomonas aeruginosa.*** PAO1 WT (*A* and *C*) and *Escherichia coli* UTI 189 (*B* and *D*). Treatment was carried out in 1× PBS media (*A* and *B*) or M9 glucose media (*C* and *D*). Both planktonic and biofilm cells were collected from the same sample well, with planktonic cells (*black circles*) referring to suspended cells present in treatment buffer prior to washing and collection of biofilm cells (*open blue triangles*). No significant differences were observed between TBAJ-5307 and untreated controls. At least two independent experiments were carried out, each with two technical replicates. Each data point represents averaged data of two independent experiments with three technical replicates, and the *line* represents the means of all data. Colony-forming unit (CFU) counts were analyzed using GraphPad Prism, version 9.3.0 using two-way ANOVA and multiple comparison of column effect (concentration of compound against untreated control) within each row (planktonic *versus* biofilm samples).

To further confirm TBAJ-5307’s target specificity and to demonstrate that the molecule does not affect biofilm formation or planktonic bacteria, the compound was assayed against *Pseudomonas aeruginosa* PAO1 or *Escherichia coli* UTI 189. As revealed in Figure 4, no killing of planktonic cells or biofilm cells was observed upon addition of 9× and 26× MIC_50_ (40 and 120 nM) of TBAJ-5307 to batch biofilms of *P. aeruginosa* PAO1 or *E. coli* UTI 189 under both nutrient-limited conditions, which was simulated by treatment in PBS, and under growth conditions, which is simulated by addition of fresh growth media. These results confirm TBAJ-5307’s target specificity and underlines that the compound is not a broad-spectrum antibiotic.

### *Ex vivo* anti-*Mab* potency of TBAJ-5307

**Figure 5:**
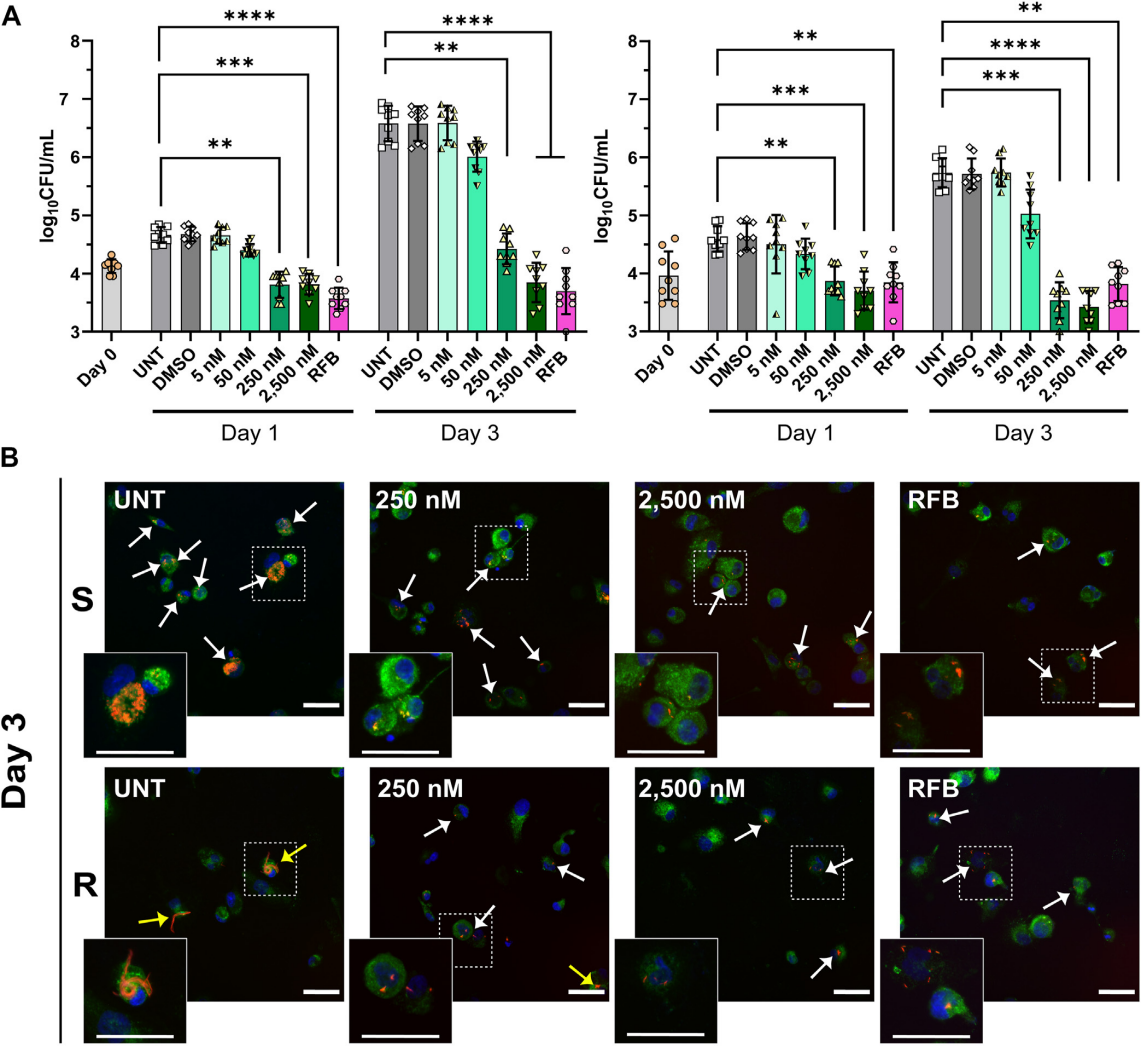
**Intracellular activity ofTBAJ-5307 against*****Mab*****CIP104536**^**T**^**infected THP-1 cells.***A* Macrophages were infected with *Mab* smooth (*left*) or rough (*right*) (MOI of 2:1) for 4 h prior to treatment with TBAJ-5307 (5, 50, 250, or 2500 nM), rifabutin (RFB at 12.5 μg/ml), DMSO, or untreated. Colony-forming units (CFUs) were determined at 0, 1, and 3 days after infection. Data are mean values ± SD for three independent experiments analyzed using Kruskal–Wallis with Dunn’s multiple comparisons test. ∗∗*p* ≤ 0.01; ∗∗∗*p* ≤ 0.001; ∗∗∗∗*p* ≤ 0.0001. *B*, representative immune-fluorescent fields were taken at 3 days postinfection showing macrophages infected with *Mab* expressing Tdtomato (*red*) in the absence of antibiotics (UNT) or in the presence of TBAJ-5307 (250 nM or 2500 nM) and RFB (12.5 μg/ml). Scale bar represents 50 μm. The surface and the endosomal system of the macrophages were detected using anti-CD63 antibodies (*green*). The nuclei were stained with 4′,6-diamidino-2-phenylindole (DAPI; *blue*). *White arrows* indicate bacilli, and *yellow arrows* indicate mycobacterial cords. Enlargement scale bar represents 50 μm. DMSO, dimethyl sulfoxide; *Mab*, *Mycobacterium abscessus*; MOI, mmultiplicity of infection.

To explore anti-*Mab* potency of the compound in macrophages, a THP-1 infection model was used. Macrophages were infected with *M*. subsp. *abscessus* S and R variants and treated with TBAJ-5307 in a range of 5 nM to 2500 nM. Figure 5*A* demonstrates that TBAJ-5307 was active against both morphotypes, reflected in decrease in viable bacterial count (colony-forming unit/ml) when compared with initial inoculum at 250 and 2500 nM. Drug-treated macrophages did not show any visual alteration of the membrane, cell form, or size as tested microscopically (Fig. 5*B*). Immunofluorescence imaging also showed reduced numbers of infected macrophages and cords (for *Mab* R) during treatment with TBAJ-5307 (Fig. 5*B*), as reported earlier with RFB (16, 17), in agreement with the reduced intracellular bacterial burden (Fig. 5*A*).

### *In vivo* potency and nontoxicity of TBAJ-5307

**Figure 6:**
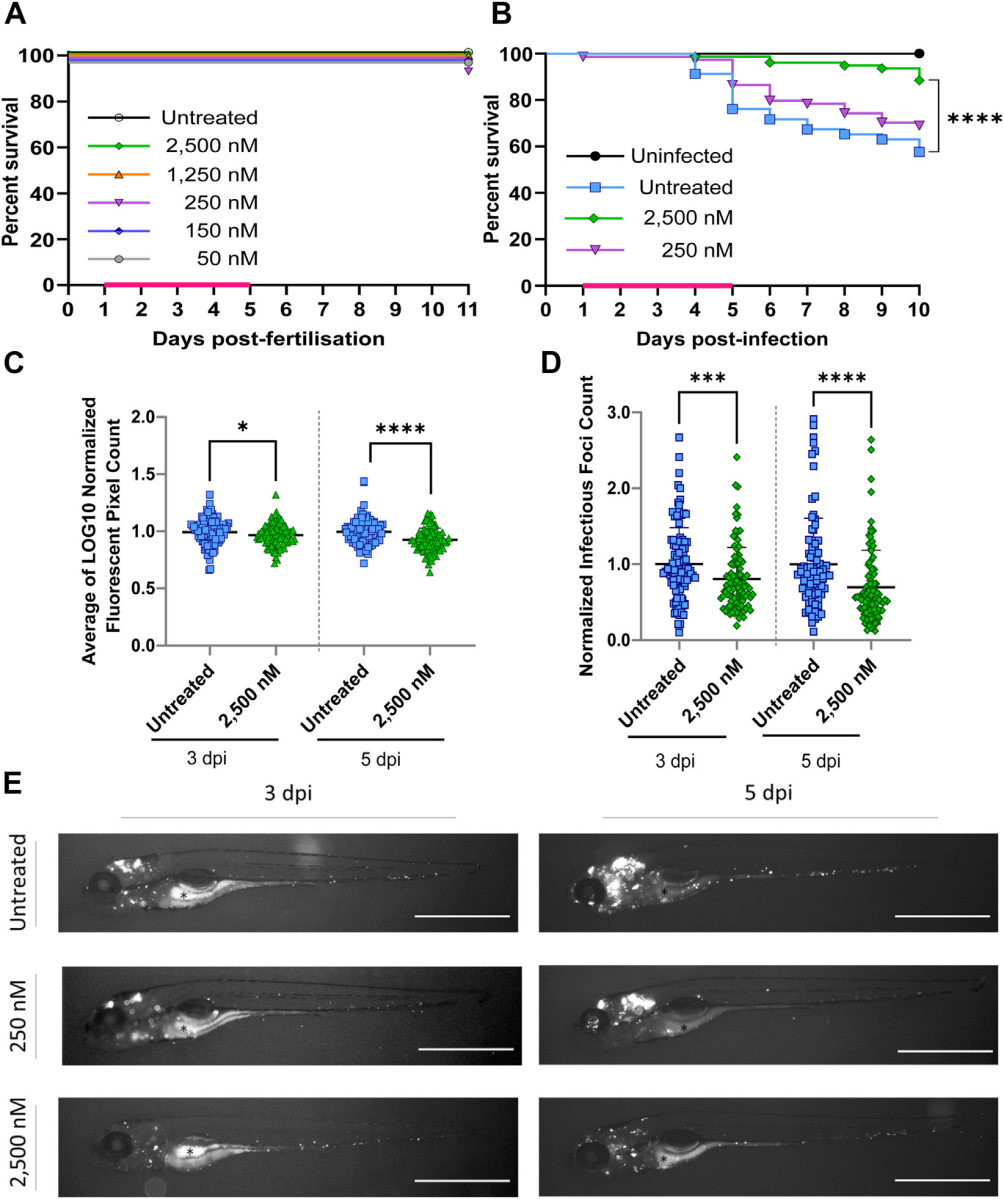
**Therapeutic activity of TBAJ-5307 against *Mab subsp. abscessus* (R variant) in an embryonic zebrafish infection model.***A*, groups of around 20 uninfected embryos were immersed in water containing increasing concentrations of TBAJ-5307 (ranging from 50 to 2500 nM) for 4 days. The *magenta bar* indicates the duration of treatment. The graph shows the survival of the TBAJ-5307-treated and -untreated embryos over an 11-day period. *B*, zebrafish embryos (around 35 per group) at 30 h postfertilization were infected with ∼250 colony-forming unit (CFU) of *Mab subsp. abscessus* expressing red fluorescent TdTomato *via* caudal vein injection. A standard PBS injection control (uninfected) was included. At 1 day postinfection, embryos were exposed to increasing concentrations of TBAJ-5307 (250 or 2500 nM) in fish water. Drugs were renewed at a daily basis for 4 days (*magenta bar*) after which embryos were washed twice in fresh embryo water and maintained in fish water. Survival was monitored daily over 11-day period. Each treatment group was compared against the untreated control group with significant differences determined using the log rank (Mantel–Cox) statistical test for survival curves: ∗∗∗∗*p* < 0.0001; *ns*, nonsignificant. Three experiments were performed. *C*, *Mab* infection burden was quantified by fluorescent pixel count (FPC) determination using the ImageJ software after 2 (3 dpi) or 4 (5 dpi) days of exposure to TBAJ-5307 (2500 nM). The data are from three experiments (containing around 35 embryos per group), with each data point representing one infected zebrafish larva. The error bar represents the mean and standard deviations of the dataset. Statistical comparison of the difference using a Mann–Whitney’s *t* test: ∗*p* < 0.05, ∗∗∗∗*p* < 0.0001. *C* and *D*, the 2500 nM values are normalized to the values of the untreated control at each time point. *D*, *Mab* infection foci in embryos after 2 (3 dpi) or 4 (5 dpi) days of exposure to TBAJ-5307 (2500 nM) by FPC determination using the ImageJ software. The error bar represents the mean and standard deviations of the dataset. Statistical comparison was done using a Mann–Whitney’s *t* test: ∗∗∗*p* < 0.001 and ∗∗∗∗*p* < 0.0001. *E*, representative embryos from the untreated group (*upper panel*) and from the group treated with 250 or 2500 nM TBAJ-5307 at 3 dpi (*left panels*) or 5 dpi (*right panels*). Scale bars represents 300 μm. ∗ highlights autofluorescence. dpi, day postinfection; *Mab*, *Mycobacterium abscessus*.

*In vivo* potency and toxicity of TBAJ-5307 has been studied in the zebrafish model of infection, which have arisen as alternative because of the advantages of rapid development, easy and fast chemical administration, visualizing, high-throughput capabilities, and the possibility to study the dynamics of the infection in the presence of the active compound (6). By analogy with the protocol developed for BDQ (6), the activity of TBAJ-5307 against *Mab* infection in zebrafish larvae was tested using red fluorescent tdTomato-expressing *M*. subsp. *abscessus* (R variant), which was injected in the caudal vein of embryos at 30 h postfertilization and transferred to 24-well plates. TBAJ-5307 was then directly added at 1-day postinfection (dpi) to the water containing the infected zebrafish, and the TBAJ-5307 supplemented water was then changed daily for 4 days. Noninfected embryos were first exposed to increasing concentrations of TBAJ-5307 and observed under a microscope. No signs of toxicity-induced killing or developmental abnormalities were recorded in the range of concentrations tested (Fig. 6*A*). When infected embryos were exposed for 4 days to 2500 nM TBAJ-5307, a significant increased survival rate (80%) was observed compared with the untreated group of embryos (Fig. 6*B*). We next monitored the kinetics of the bacterial burden upon intravenous infection of embryos by quantification of the total area of the red fluorescent signal (fluorescent pixel count) at 3 and 5 dpi in the presence or the absence of TBAJ-5307. The addition of 2500 nM TBAJ-5307 for 4 days in fish water was accompanied by a significant reduction in the fluorescent pixel count at both time points (Fig. 6*C*). Importantly, our results indicate that at both 3 and 5 dpi, treatment with TBAJ-5307 coincided with a significant reduction in the number of infection foci (Fig. 6*D*). Whole embryo imaging at 3 and 5 dpi clearly highlighted the effect of the compound, with limited infection, particularly evident in the presence of 2500 nM TBAJ-5307 (Fig. 6*E*), consistent with bacterial burden quantification (Fig. 6*C*). This pronounced effect of the compound in reducing the bacterial burden and infection foci is likely to explain the protective efficacy of TBAJ-5307 on embryo survival. Abscess formation can be linked to loss of infection control, typically occurring following cord formation and expansion and representing a marker of disease severity (18, 19, 20), associated with cell debris, tissue destruction, and acute infection. We note that treatment with TBAJ-5307 was associated with a decrease in abscess formation and cording (Figs. 6*E* and S5). This indicates that TBAJ-5307 is very efficient *in vivo* against infection and protects the zebrafish from killing by the pathogen *M*. subsp. *abscessus*.

### TBAJ-5307 enhances potency of existing anti-*Mab* drug candidates

**Figure 7:**
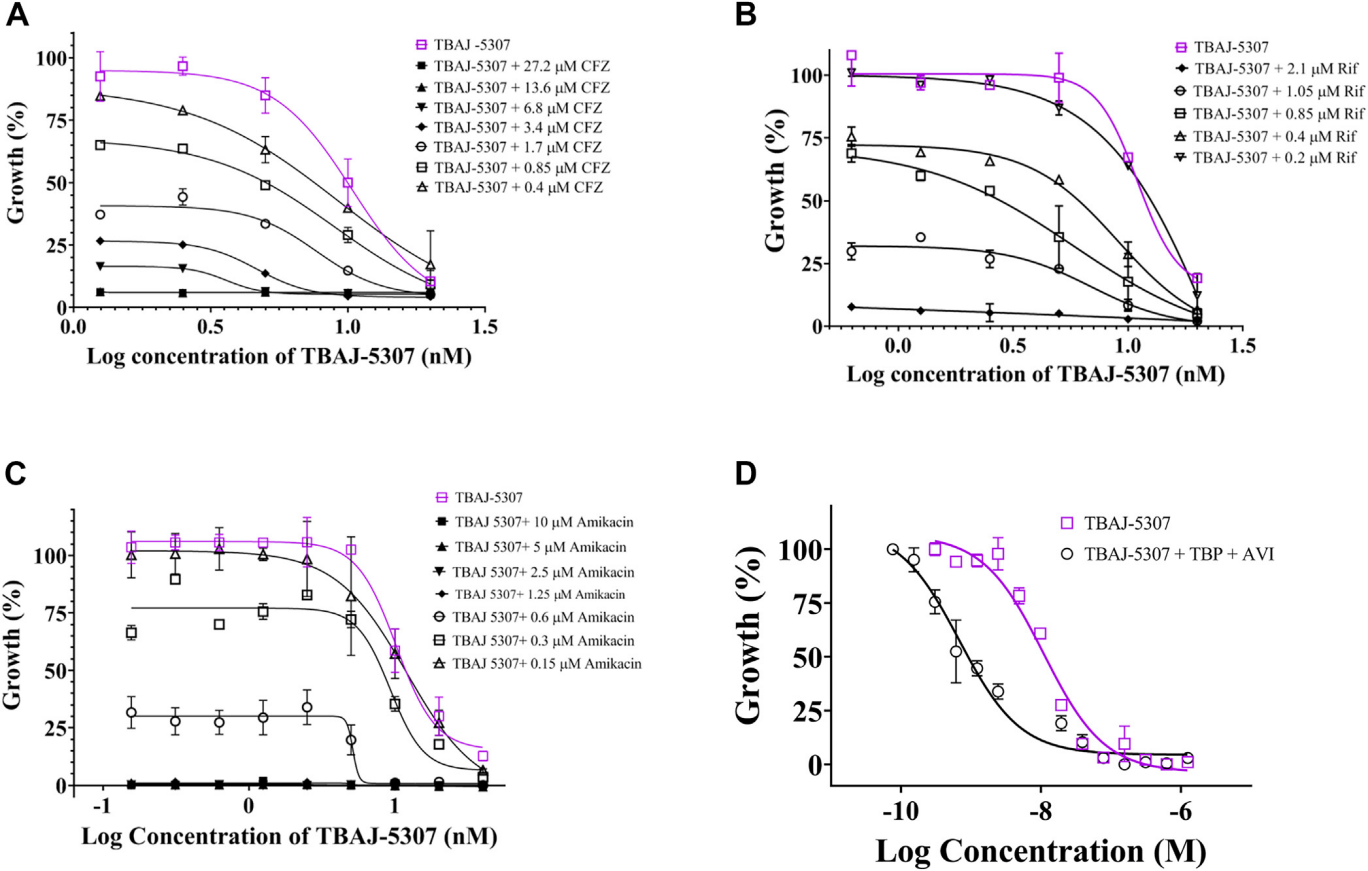
**Increased potency of TBAJ-5307 in combination with the antibiotics CFZ, rifabutin, and amikacin in 7H9 broth.***Mab* subsp. *abscessus* growth inhibition by TBAJ-5307 in combination with increasing concentrations of CFZ (*A*), rifabutin (*B*), and amikacin (*C*). ∗∗∗∗*p* < 0.0001, statistical analysis was carried out using two-way ANOVA test for all the experiments presented. *D*, growth inhibition dose–response of TBAJ-5307 alone and in combination of TBAJ-5307 with TBP and AVI against Mab ATCC 19977. TBAJ-5307 alone showed an MIC_50_ of 10 nM. In comparison, its MIC_50_ (0.7 nM) was 14 times lower in combination with TBP (4 μM) plus AVI (14 μM). ATCC, American Type Culture Collection; AVI, avibactam; CFZ, clofazimine; *Mab*, *Mycobacterium abscessus*; MIC_50_, minimum inhibitory concentrations; TBP, tebipenem.

Since leveraging the potential of synergistic drug interactions in multidrug treatment regimens to accelerate durable cure is a growing field and the treatment of *Mab* infections requires drug combinations, the growth inhibition activity of TBAJ-5307 along with clinical *Mab* antibiotics CFZ, amikacin, and the RNA polymerase inhibitor, RFB, was measured using a checker-board titration assay. Drastic reduction of cell growth was observed with all three drugs tested (Fig. 7, *A*–*C*). The fractional inhibitory concentration index was calculated to characterize the interaction between TBAJ-5307 and each of the test drugs (21). Fig. S6, *A*–*C* shows an additive growth reduction triggered by CFZ or RFB with TBAJ-5307, achieving a fractional inhibitory concentration index of 0.75 and 0.77, respectively in *M*. subsp. *abscessus*. The results demonstrate that TBAJ-5307 enhances the potency of major antibiotics against *Mab*. Treatment of *Mab* lung diseases by the aminoglycoside amikacin or the β-lactams imipenem and cefoxitin displays modest *in vitro* activity and is administered by the intravenous route. Given the expense and risks of intravenous therapies, more potent oral combinations would provide huge advantages. Recently, the combination of the oral β-lactam TBP, targeting the l,d-transpeptidases LdtMab1 and LdtMab2 and d,d-transpeptidases PonA1, PonA2, and PbpA, which are essential for peptidoglycan synthesis, with the *Mab* β-lactamase inhibitor AVI has shown an attractive, low micromolar, and strong cidal activity (Fig. S6*D*) (22, 23, 24). Here, we tested whether inhibiting the fundamental cell processes of peptidoglycan synthesis in cell wall formation and silencing of the essential mycobacterial enzyme F-ATP synthase by TBAJ-5307 provide a potent combination. As shown in Figure 7*D*, combining the TBP–AVI pair with TBAJ-5307 enhanced growth inhibition. The efficacy of TBAJ-5307 increased by a factor of 14 (MIC_50_ of 0.7 nM) in combination of TBP–AVI. Importantly, TBAJ-5307 does not exert any observable antagonism in killing activity with bactericidal TBP–AVI (Fig. S6*E*), suggesting that TBAJ-5307 could be coadministered with the dual drug.

### TBAJ-5307 is a broad spectrum anti-NTM antibiotic

**Figure 8:**
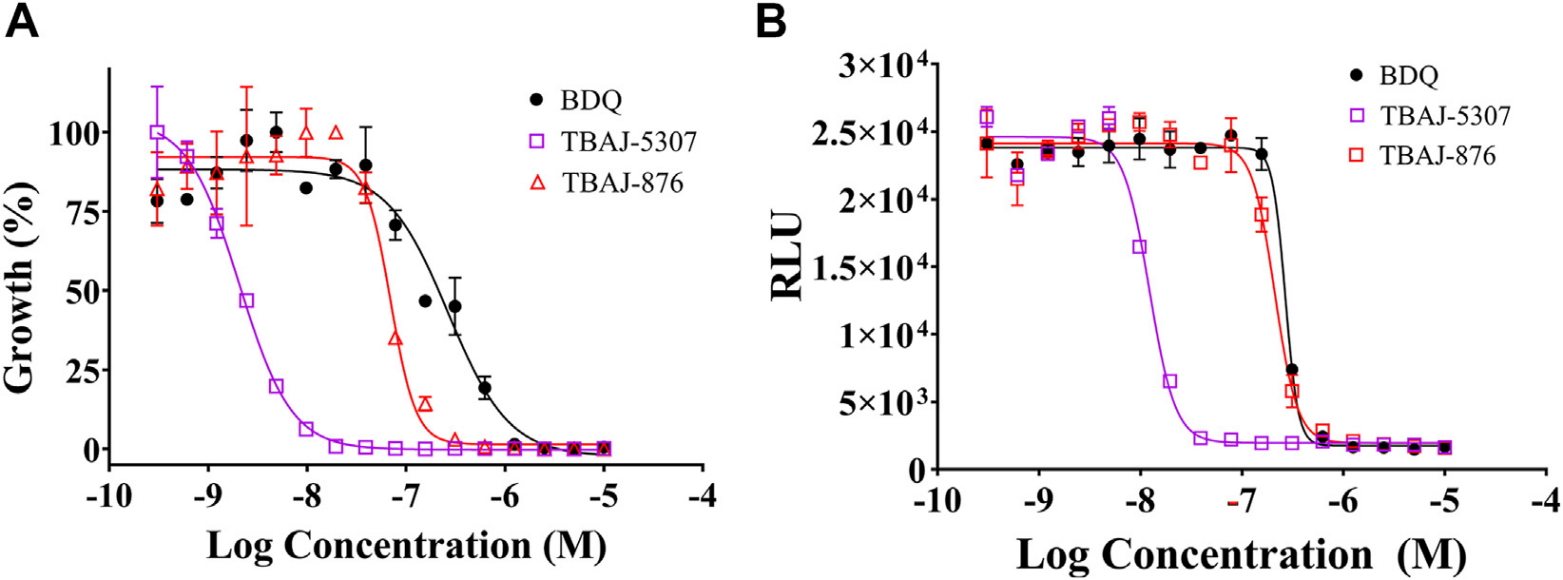
**Anti-*M. avium* potency of TBAJ-5307.***A*, growth inhibition dose–response curve of *Mycobacterium avium* by TBAJ-5307 in comparison to BDQ and TBAJ-876. *B*, TBAJ-5307 inhibits oxidative phosphorylation in a whole-cell ATP synthesis assay better than BDQ or TBAJ-876. Three biological replicates were carried out each in three technical replicates. Data represent the average of all the experiments. ∗∗∗∗*p* < 0.0001, statistical analysis was carried out using two-way ANOVA test for all the experiments presented. BDQ, bedaquiline.

We also explored TBAJ-5307’s efficacy against the NTM fast growers *M. mucogenicum* and *M. fortuitum* as well as against the slow growers *M. avium* and *M. intracellulare*. Together with the *M. abscessus* complex, the *Mycobacterium avium* complex, including *M. avium* and *M. intracellulare*, are responsible for most cases of pulmonary infections worldwide (4). Here, we reveal that TBAJ-5307 is highly potent against *M. avium* with an MIC_50_ value of 1.8 ± 0.2 nM, which is 100 and 36 times better than BDQ (184 ± 23 nM) and TBAJ-876, respectively (64 ± 4.8 nM; Table 1 and Fig. 8*A*). It also inhibits growth of the clinical isolate *M*. avium (25) with an MIC_50_ of 31 ± 2.8 nM (Fig. S7*A* and Table 1). Whole-cell ATP synthesis studies confirmed that growth inhibition of TBAJ-5307 directly correlated with depletion of ATP and that TBAJ-5307 (IC_50_ = 12 ± 4 nM) is the strongest inhibitor, when compared with BDQ (IC_50_ = 267 ± 30 nM) and TBAJ-876 (IC_50_ = 210 ± 25 nM) (Table 1 and Fig. 8*B*). TBAJ-5307 showed slightly better growth inhibition against *M. intracellulare* (16 ± 3.2 nM) when compared with BDQ (60 ± 2.8 nM) and TBAJ-876 (30 ± 2.5 nM) (Table 1 and Fig. S7*B*), which is caused by the stronger inhibitory effect of TBAJ-5307 in ATP formation (6.7 ± 2.1 nM; Table 1 and Fig. S7*C*).

Growth and whole-cell ATP synthesis of the fast grower *M. mucogenicum*, causing respiratory-related, central nervous–related, catheter-related-, skin and soft tissue infections (1), was inhibited by TBAJ-5307 with an MIC_50_ of 1.2 nM ± 0.4 nM and an IC_50_ of 3.1 ± 0.9 nM, respectively (Fig. S7, *D* and *E*). As displayed in Fig. S7*F*, TBAJ-5307 is also potent against *M. fortuitum* (MIC_50_ values of 0.9 ± 0.1 nM), which is mainly isolated from patients with skin and soft tissue infection (26), and appears slightly more potent compared with TBAJ-876 (1.7 ± 0.2 nM) or BDQ (8.3 ± 7 nM).

The data demonstrate that TBAJ-5307 is a highly potent anti-NTM agent covering the broad range of fast and slow NTM growers by targeting the F_1_F_O_-ATP synthase, which converts ADP and Pi to the biological currency ATP. The data also show, for the first time, that TBAJ-876, reported to be active against the *M. abscessus* complex (9), displays attractive *in vitro* activity against *M. mucogenicum*, *M. fortuitum*, *M. avium*, and *M. intracellulare*, although with lower potency compared with TBAJ-5307.

## Conclusion

NTM infection presents a growing global health problem, complicated by ubiquitous exposure to the organisms, incomplete understanding of the immune susceptibility to disease, increasing numbers of immune-compromised patients, and costly multidrug treatment regimens that often fail to cure. Therefore, there is a pressing need to identify inhibitors with high efficacy and enzyme targets being essential for NTM under various metabolic conditions, being nontoxic and with attractive combinatory potency. Here, we identified TBAJ-5307 as a novel anti-*Mab* inhibitor targeting the F-ATP synthase, essential for ATP synthesis, regulation of ATP homeostasis, and proton motive force under multiple growth conditions. Based on our MD simulations and free-energy calculations, the inhibitor interacts with the F_O_ domain *via* the *c*-ring as well as the subunits *a*–*c* lagging and leading sites, including the *Mab*-specific residues *a*F177, *a*I181, *a*A218, and *a*I222 (Fig. 3). TBAJ-5307’s binding to the *a*–*c* interfaces inhibits proton translocation *via* the F_O_ domain half-channels by preventing rotation of the *c*-ring relative to subunit *a*. The high potency of TBAJ-5307 in growth inhibition correlates with whole-cell ATP depletion, reduction in ATP formation of *Mab* inverted-membrane vesicles, and free-energy calculations (Fig. 2). The compound displays higher efficacy compared with BDQ and TBAJ-876. TBAJ-5307 exhibits anti-*Mab* potency in macrophages (Fig. 5) and zebrafish embryos without being toxic (Fig. 6), which is essential for further clinical developments. The compound potentiates the anti-*Mab* activity of the NADH dehydrogenase inhibitor CFZ, the 16S rRNA targeting amikacin, the RNA polymerase–targeting antibiotic RFB, and the oral pair TBP–AVI, with TBP inhibiting peptidoglycan synthesis, respectively (Fig. 7). In addition, the TBAJ-5307–TBP–AVI cocktail is bactericidal (Fig. S6*E*). The presented combinations of the low nanomolar ATP synthesis inhibitor TBAJ-5307 with antibiotics targeting transcription, protein synthesis, or cell wall formation would be a step forward (i) to silence major cell processes of the pathogen under different metabolic states, (ii) to lower the emergence of drug resistance, (iii) to reduce the required concentrations and thus dosing of these existing antibiotics because of the enhanced inhibitory activity of each within the cocktail, leading to reduced toxicity and side effects, and (iv) in case of an TBAJ-5307–TBP–AVI to aim for an oral drug combination, which would reduce costs and risks compared with intravenous therapies. Importantly, TBAJ-5307 displays pronounced nanomolar anti-NTM activity, which correlates with whole-cell ATP depletion, underscoring binding of TBAJ-5307 to the NTM F_1_F_O_-ATP synthase (Fig. 8). Its improved potency compared with BDQ and TBAJ-876 makes the compound an attractive candidate to eradicate a broad range of NTM infectious disease in future.

## Experimental procedures

### Synthesis of TBAJ racemates (±)-5307, (±)-5316, and (±)-5366 and TBAJ-5307

The racemates (±)-5307, (±)-5316, and (±)-5366 were synthesized according to Hotra *et al.* (12). Chiral separation service of (±)-5307 was provided by WuXi AppTec (Wuhan) Co, Ltd. The desired chiral isomers were separated by ChiralPAK AD-3 column using supercritical fluid chromatography with gradients of isopropanol/CO_2_/diethanolamine as eluent. Purity and enantiomeric excess of the two enantiomers of TBAJ-5307 were >94% and >99%. The levorotatory enantiomer showed highest activity and was assigned (1*R*, 2*S*) stereochemistry in accordance with Sutherland *et al.* (11). LC–MS *m/z* [M + H]^+^ 627.4. [α]_D_ (25) = −6.28 (c = 0.1, CHCl_3_) (11).

### BDQ and its 3,5-dialkoxypyridine analog TBAJ-876

BDQ was purchased from MedChem Express, and TBAJ-876 was synthesized as described by Sarathy *et al.* (9). Both inhibitors were dissolved in 100% dimethyl sulfoxide (MP Biomedicals).

### Zebrafish ethics statements

All zebrafish experiments were approved by the Direction Sanitaire et Vétérinaire de l’Hérault for the ZEFIX-CRBM zebrafish facility (Montpellier) (registration number: C-34-172-39). Handling and experiments were approved by “le ministère de l’enseignement supérieur, de la recherche et de l’innovation” under the reference APAFIS#24406-2020022815234677 V3.

## Data availability

All relevant data are contained within the article.

## Supporting information

This article contains supporting information (10, 12, 13, 15, 20, 21, 25, 27, 28).

## Conflict of interest

G. G., R. W. B., P. R., P. S.-L., and L. K. are inventors on the patent 23306555.6, which is related to the inhibitor described in this article. All other authors declare that they have no conflicts of interest with the contents of this article.
