# Supplementary material for: High efficacy of the F-ATP synthase inhibitor TBAJ-5307 against nontuberculous mycobacteria in vitro and in vivo
Source: J Biol Chem. 2024 Jan 3;300(2):105618. doi: 10.1016/j.jbc.2023.105618 (PMC10840338; doi:10.1016/j.jbc.2023.105618)
Supplement: Supporting information [file mmc1.pdf]

High efficacy of the F-ATP synthase inhibitor TBAJ-5307 against non-tuberculous mycobacteria *in vitro* and *in vivo*

Priya Ragunathan[1], Patcharaporn Sae-Lao[2], Claire Hamela[3], Matthéo Alcaraz[3], Alexander Krah[4], Wee Han Poh[5], Carmen Jia Ern Pee[6], Albert Yick Hou Lim[6,7], Scott A. Rice[1,4,8], Kevin Pethe[1,4,6,9], Peter John Bond[4], Thomas Dick[10-12], Laurent Kremer[3,13,*], Roderick W. Bates[2,*], and Gerhard Grüber[1,4*]

[1]School of Biological Sciences, Nanyang Technological University, 60 Nanyang Drive, Singapore 637551; E-mail: ggrueber@ntu.edu.sg
[2]School of Chemistry, Chemical Engineering and Biotechnology, Nanyang Technological University, 21 Nanyang Link, Singapore 637371; E-mail: Roderick@ntu.edu.sg
[3]Centre National de la Recherche Scientifique UMR 9004, Institut de Recherche en Infectiologie de Montpellier (IRIM), Université de Montpellier, 1919 route de Mende, 34293, Montpellier, France; E-mail: Laurent.kremer@irim.cnrs.fr
[4]Bioinformatics Institute, Agency for Science, Technology and Research (A*STAR), 30 Biopolis Str., #07-01 Matrix, Singapore 138671
[5]Singapore Centre for Environmental Life Sciences Engineering, Nanyang Technological University, Singapore 637551
[6]Lee Kong Chian School of Medicine, Nanyang Technological University, Experimental Medicine Building, Singapore 636921
[7]Department for Respiratory and Critical Care Medicine, Tan Tock Seng Hospital, Singapore 308433
[8]Microbiomes for One Systems Health and Agriculture and Food, CSIRO, Westmead NSW, Australia
[9]National Centre for Infectious Diseases (NCID), 16 Jalan Tan Tock Seng, Singapore 308442
[10]Center for Discovery and Innovation, Hackensack Meridian Health, 340 Kingsland Street, Nutley, NJ 07110, USA
[11]Department of Medical Sciences, Hackensack Meridian School of Medicine, 123 Metro Boulevard, Nutley, NJ 07110, USA
[12]Department of Microbiology and Immunology, Georgetown University, 3900 Reservoir Road NW Medical-Dental Building, Washington, DC 20007, USA
[13]INSERM, IRIM, 34293 Montpellier, France.

Running title: Potent anti-NTM inhibitor targeting ATP formation

Keywords: Mycobacteria; Non-tuberculosis mycobacterium; antibiotics; bacterial pathogenesis; membrane protein; ATP synthesis

Table of Contents

1. **Biological procedures and data**
   - Bacterial strains and culture media S3
   - Growth inhibition dose-response assay S3
   - Quantification of intracellular ATP levels S4
   - Preparation of *M. abscessus* subsp *abscessus* inverted membrane vesicles S4
   - ATP synthesis assay to determine ATP formation of *M. abscessus* IMVs S4
   - Checker-board titration assay S5
   - Bacterial killing assay S6
   - Methylene blue assay S6
   - Biofilm testing S7
   - Macrophage experiments S7
   - Zebrafish infection experiments S8
   - Zebrafish care and ethics statements S9
   - Microscopy and image analysis S9

2. **Computational procedures and data**
   - Conventional MD simulations S10
   - Free energy calculations S10

3. **Supplementary Figures**
   - Supplementary Figure 1 S12
   - Supplementary Figure 2 S12
   - Supplementary Figure 3 S13
   - Supplementary Figure 4 S15
   - Supplementary Figure 5 S16
   - Supplementary Figure 6 S17
   - Supplementary Figure 7 S19

4. **Supplementary Tables**
   - Supplementary Table 1 S20

5. **References** S21

1. Biological procedures and data

Bacterial strains and culture media

The NTM strains *M. abscessus subsp. abscessus*, *M. abscessus subsp. bolletii*, *M. abscessus subsp. massiliense*, *M. mucogenicum*, *M. fortuitum*, *M. avium* and *M. intracellulare,* and clinical isolates strains *M. abscessus* Bamboo, *M. avium* 11, *M. abscessus subsp. massiliense* clinical isolate 1 and *M. abscessus subsp. massiliense* clinical isolate 2 were used. The clinical isolate *M. abscessus* Bamboo has been isolated from a patient with amyotrophic lateral sclerosis and bronchiectasis, belongs to the subspecies *M. abscessus* subsp. *abscessus* and harbors the inactive, clarithromycin-sensitive *erm*(41) C28 sequevar (GenBank accession no. MVDX00000000) (1). The clinical isolate *M. avium* has been isolated from bone marrow of an AIDS patient with disseminated MAC infection, including pulmonary infection, and it is classified as *M. avium subsp. hominissuis* based on the 3′ region of the *hsp65* gene sequence with 100% identity when aligned with the *hsp65* 3′ region of *M. avium* subsp. *hominissuis* 104 (GenBank accession no. NC_008595) (2). *M. massiliense* clinical isolate 1 and *M. massiliense* clinical isolate 2 were isolated from the sputum of patients with NTM pulmonary infection and subspecies classification was determined by PCR of *erm*(41) which produced a truncated 397 bp product (3). All *M. abscessus* strains were maintained in Middlebrook 7H9 medium (BD Difco) supplemented with 0.2% (vol/vol) glycerol (Fisher Scientific), 0.05% (vol/vol) Tween 80 (Sigma-Aldrich), and 10% (vol/vol) Middlebrook albumin-dextrose-catalase (ADC) (BD Difco).

Growth inhibition dose-response assay

Growth inhibition dose-response assay was carried out by the broth microdilution method (4). Briefly, each well of clear 96-well flat-bottom Costar cell culture plates (Corning) was filled with 100 µl of liquid medium (complete 7H9 medium). TBAJ-5307/ TBAJ-876/BDQ were added to the first well in each row of the plate to create two times the desired highest final concentration. A 10-point 2-fold serial dilution of TBAJ-5307/TBAJ-876/BDQ was carried out starting from this first well. The *M. abscessus* strains used for this assay were grown to mid-exponential phase and then diluted to an optical density at 600 nm (OD_{600}) value of 0.1 using the same liquid medium used to run the assay. One hundred microliters of the diluted culture were added to each well to create a final OD_{600} value of 0.05 in each well. The plates were incubated for 3 days at 37 °C. After the

incubation period, the cultures in all wells were manually resuspended, and the OD_{600} of each well was read using a Tecan Infinite Pro 200 plate reader. The reported MIC_{50} values represent the concentration that inhibits 50% of bacterial growth compared to the untreated culture.

Quantification of intracellular ATP levels

100 µl of complete 7H9 medium were filled in each well of clear 96-well flat-bottom Costar cell culture plates (Corning). TBAJ-5307/TBAJ-876/BDQ was added to the first well in each row to create two times the desired highest final concentration. Subsequently, a 16-point 2-fold serial dilution was carried out starting from the first well in each row. The respective *M. abscessus* strain, which was grown to mid-exponential phase, was diluted to an OD_{600} of 0.1; 100 µl of the diluted culture was added to each well to create a final OD_{600} of 0.05 in all the wells. The plates were incubated at 37 °C for 6-8 h.

At the end of the incubation period, the samples were measured for their intracellular ATP content by adding the BacTiter-Glo microbial cell viability assay (Promega), which was carried out according to the manufacturer's instructions as described previously (4, 5). 50 µl of bacterial sample was mixed with 50 µl of the BacTiter-Glo reagent in each well of an opaque, white, 96-well, flat-bottom Nunc plate. Luminescence was measured with a Tecan Infinite Pro 200 plate reader after 10 min of incubation of the plate in the dark at room temperature. The background luminescence reading was subtracted from the luminescence readings of all the samples. The amount of ATP content is directly proportional to the relative luminescence units. The graph of the results was made using GraphPad Prism 8 software (6).

Preparation of *M. abscessus* subsp *abscessus* inverted membrane vesicles

Inverted membrane vesicles (IMVs) of *M. abscessus subsp. abscessus* R variant were prepared according to a method described previously to Hotra et al. (7).

ATP synthesis assay to determine ATP formation of *M. abscessus* IMVs

The reaction mixture for the ATP synthesis assay contained assay buffer (50 mM MOPS [morpholinepropanesulfonic acid]-NaOH, 10 mM $MgCl_2$, pH 7.5), 10 µM ADP, 250 µM P_i, and 1 mM NADH. KH_2PO_4 salt (100 mM) was dissolved in the assay buffer to adjust the concentration

of inorganic phosphate (P_i); 25 µl of the reaction mixture was added to each well of an opaque, white, 96-well, flat-bottom Nunc plate (Thermo Scientific). TBAJ-5307 was added to the first well of each row to create 2x the desired highest final concentration. A 16-point 2-fold serial dilution was then carried out starting from the first well. *M. abscessus subsp. abscessus* IMVs were then added to create a final concentration of 5 µg of protein/ml in 50 µl per well. Subsequently, the plate was incubated at room temperature for 30 min. At the end of the incubation period, 50 µl of CellTitre-Glo (Promega) was added to each well. The plate was then incubated again for 10 min in the dark at room temperature. Subsequently, luminescence was measured with a Tecan Infinite Pro 200 plate reader (parameters: luminescence, integration time of 500 ms; no attenuation). The level of luminescence correlates with the amount of ATP synthesized by the F-ATP synthase. The graphs of the results were made using GraphPad Prism 8 software (6).

Checker-board titration assay

A checkerboard titration assay was carried out as described previously (8, 9). Briefly, TBAJ-5307 and CFZ/Rifabutin/Amikacin were added to complete 7H9 medium-containing 96-well flat-bottom Costar cell culture plates. Two-fold serial dilutions were done to allow 10 different concentrations of TBAJ-5307 (40 nM to 0.03 nM) to be tested for interaction with 7 different concentrations of CFZ (27.2 µM to 0.2 µM). Hence, a total of 70 different concentration combinations were tested between TBAJ-5307 and CFZ. In combination studies with Rifabutin and Amikacin, 10 different concentrations of TBAJ-5307 (40 nM to 0.03 nM) were tested with 7 different concentrations of Rifabutin (18.8 µM to 0.2 µM) and Amikacin (10 µM to 0.15 µM). Each 96-well plate had a 7H9 medium-only control well and a drug-free bacterial culture control well. *M. abscessus subsp. abscessus* R variant was cultured in complete 7H9 medium and grown to mid-exponential phase. Subsequently, the culture was diluted to an OD_{600} of 0.01 using complete 7H9 medium and added to each well in the 96-well plate to create a final OD_{600} value of 0.005. The plates were incubated for 4 days at 37 °C. After the incubation period, the culture in each 96-well plate was manually resuspended, and the OD_{600} of each well was read using a Tecan Infinite Pro 200 plate reader.

In order to assess the combined effect of TBAJ-5307 and TBP/AVI, we performed checkerboard analyses using *M. abscessus* ATCC 19977 and a 96-well plate format was employed. The optical

density at 600 nm (OD600) was measured as an indicator of growth. We evaluated the impact of varying concentrations of TBP in a serial dilution, spanning from 12.5 to 0.012 µM, in combination with a fixed concentration of 14 µM AVI. TBAJ-5307 were tested at doses ranging from 10 µM to 0.01 nM.

Calculation of the fractional inhibitory concentration index (FICI) was done to analyze the results. The FICI is calculated as (MIC of drug A in combination/MIC of drug A alone) + (MIC of DARQ B in combination/MIC of DARQ B alone). This calculation was done only for wells which showed 50% inhibition of bacterial culture growth compared to drug-free bacterial culture wells. A FICI of ≤0.5 indicates synergy, a FICI of >0.5 to 4 indicates additivity (no interaction), and a FICI of >4 indicates antagonism (10).

Bacterial killing assay

M. abscessus subsp. *abscessus* culture were grown to exponential phase and diluted to an OD_{600} of 0.005 and aliquoted onto T-25 mm^2 tissue culture flasks. Test compounds were dispensed into each flask and were incubated at 37 °C for 5 days. Around 10 µl of culture was taken out from each flask followed by the serial dilution with Phosphate-buffered saline (PBS). 25 µl of culture of respective dilutions were plated on each quadrant of 7H10 agar plate. The agar plates were incubated at 37 °C for five days. Bacterial viability was determined by counting the colony-forming units (CFU) on the plates.

Methylene blue assay

Aliquots of 1.5 ml (OD_{600} = 0.3) log-phase cultures of *M. abscessus* subsp. *abscessus* were transferred into clear 2 ml screw-cap glass vials along with test compounds. The cultures were incubated in the presence of the drugs for 6 h at 37 °C before the addition of methylene blue dye at a final concentration of 0.001%. Upon addition of methylene blue, all vials were tightly closed and incubated at 37 °C in a hypoxic jar for 72 hours. Oxygen in the jar was removed by AnaeroGen sachet. The dye decolorizes when the oxygen in the culture is used up. The color changes and the differences in the exponentially growing cultures and broth reflect the relative oxygen consumption, thus indirectly indicate the respiration of each culture.

Biofilm testing

Biofilm assay was performed in a 24-well plate to evaluate the activity of BDQ and TBAJ-5307 on *Pseudomonas aeruginosa* PAO1 and *Escherichia coli* UTI 189 biofilms. Biofilms were tested in microtiter plates and 3 h incubation to form biofilms. This time point avoids the confounding issues of biofilm dispersal, which happen within 10 h incubation in when the carbon source is exhausted (11, 12). Overnight cultures of *P. aeruginosa* PAO1 and *E. coli* UTI 189 were diluted in M9 glucose media (1 × M9 salts, 2 mM $MgSO_4$, 0.1 mM $CaCl_2$, 0.4% w/v glucose) to a final OD_{600} of 0.05. One ml of the diluted culture was then added into each well of the 24-well plate and incubated at 37°C with 100 rpm shaking. Following 3 h of incubation, each well was washed once, and the culture media was replaced with 1 ml of 1 × PBS (phosphate buffered saline; pH 7.4) or fresh M9 glucose medium containing 1 µM of BDQ or 40 and 120 nM of TBAJ-5307, respectively. The treated samples were further incubated under the same conditions for 3 h. At t = 6 h, the samples were collected. The 1 × PBS (pH 7.4) buffer containing suspended bacteria cells with treatment was collected into 1.5 ml Eppendorf tubes and considered to be "planktonic samples". Subsequently, each well was washed once with 1 ml 1 × PBS (pH 7.4) before resuspending biofilm cells in the same volume of 1 × PBS (pH 7.4). Biofilm cells were dislodged into the buffer by means of a cell scraper and 1 ml of the sample was collected into 1.5 ml Eppendorf tubes and labelled "biofilm samples". The samples contained in Eppendorf tubes were sonicated in a water bath using the following settings: 5 min degas mode, 37 Hz, 100%, followed by 5 min pulse mode, 37 Hz, 100%. Subsequently, the samples are serially diluted and used for CFU counts. The experiment was repeated independently at least two times, with two technical replicate per independent experiment. CFU counts were analyzed using Graphpad Prism V9.3.0 using 2-way ANOVA and multiple comparison of column effect (concentration of compound against untreated control) within each row (planktonic vs biofilm samples).

Macrophage experiments

THP-1 monocytes were grown in Roswell Park Memorial Institute (RPMI) medium (BD Gibco) supplemented with 10% fetal bovine serum (FBS) (Sigma-Aldrich, Saint Quentin Fallavier, France) ($RPMI^{FBS}$) and incubated at 37 °C with 5% CO_2. Human monocytes were differentiated in macrophages with Phorbol 12-myristate 13-acetate (PMA) at 20 ng/ml for 48 hrs at 37 °C in the

presence of 5% CO_2. The intracellular activity of TBAJ-5307 was assessed on *M. abscessus* S or R-infected THP-1 cells. Each well of a 24-well microplate was seeded with 10^5 differentiated cells. Infection with each variant of *M. abscessus* was carried out at 37 °C with 5% CO_2 for 4 hrs at a MOI 2:1. After washes with PBS, an incubation of 2 hrs with 250 µg/ml of amikacin was performed to eliminate remaining extracellular bacilli prior to the addition of 500 µl of $RPMI^{FBS}$ containing either TBAJ-5307 (5 nM; 50 nM; 250 nM; 2500 nM), rifabutin (RFB; 15 µM) or DMSO (negative control). Drugs were renewed on a daily basis and lysed with 100 µl of 1% Triton X-100 after 1 day or 3 days post-infection. Colonies were counted onto LB agar plates to determine intracellular CFU.

For microscopy-based macrophages infectivity assays, THP-1 cells were prepared and treated (TBAJ-5307 -250 nM or 2500 nM-, RFB -15 µM- or not) in the same manner as described above, grown on coverslips and infected with *M. abscessus* expressing tdTomato. Cells were fixed at 3 days post-infection with 4% paraformaldehyde in 1x PBS for 20 min. Macrophages were then permeabilized using 0.2% Triton X-100 for 20 min, blocked with 2% BSA in 0.2% Triton X-100. The surface and the endolysosomal system of the macrophages were detected using anti-CD63 antibodies (Beckton Dickinson, dilution 1:1000) incubated for 1 hr and an Alexa Fluor 488-conjugated antimouse secondary antibody (Molecular Probes, Invitrogen, dilution 1:1000) incubated for 45 min. The nuclei were stained with DAPI for 5min (dilution 1:1000). Cells were then mounted on microscope slides with Immu-mount (Calbiochem) and representative immune-fluorescent fields were captured on a Zeiss Axioimager upright microscope equipped with 63x oil objective and processed using ImageJ software. The data were analyzed using the Student's unpaired two-tailed *t* test.

Zebrafish infection experiments

Experiments were done with *M. abscessus* $CIP104536^T$ (R morpphotype) expressing tdTomato, obtained after transformation with the replicative plasmid pTEC27 (13). At 24 hours post-fertilization (hpf), embryos were dechorionated manually using tweezers. At 30 hpf, embryos were anesthetized with 200 µg/ml tricaine followed by caudal vein microinjection of 3 nl of bacterial suspension (approximately 80 CFU/nl). The size of the inoculum was verified *a posteriori* by injecting 3 nl of each bacterial suspension in sterile PBS and plated on 7H10 +). To monitor embryo

survival, infected larvae were transferred into 24-well plates (four embryos/well) and incubated at 28.5 °C. Survival curves were determined by counting dead larvae at a daily basis for up to 11 days, with the experiment concluded when uninfected embryos started to die. Dead embryos were determined based on the absence of a heartbeat and removed from the well. TBAJ-5307 treatment of infected embryos and uninfected larvae was started at 1 dpi (day post-infection) for 4 days. The drug-containing solution was renewed daily and wash twice at 5 dpi. Statistical analyses were performed on Prism 9.5.1 (Graphpad, La Jolla, CA, USA) and detailed in each figure legend.

Zebrafish care and ethics statements

All zebrafish experiments were approved by the Direction Sanitaire et Vétérinaire de l'Hérault for the ZEFIX-CRBM zebrafish facility (Montpellier) (registration number C-34-172-39). Handling and experiments were approved by "le ministère de l'enseignement supérieur, de la recherche et de l'innovation" under the reference APAFIS#24406-2020022815234677 V3. Experiments were done using the *golden* mutant (14) crossed with wild-type AB zebrafish, maintained as described earlier (15).

Microscopy and image analysis

In order to visualize the infection foci, infected larvae were anesthetized in 0.02% tricaine solution as previously described (16), mounting on 3% (w/v) methylcellulose solution and taking fluorescent images using a Zeiss Axio Zoom.V16 coupled with an Axiocam 503 mono (Zeiss). Infection foci and Fluorescence Pixel Count (FPC) measurements were determined using the 'Analyse particles' function in ImageJ. Infection foci measurement is the number of fluorescent clusters per embryo (in the graph, one dot corresponds to the number of clusters in one embryo. FPC is the sum of the area of fluorescence of all the clusters per embryo. Normalization of the data was performed against the untreated control group (placed to 1). All experiments were completed at least three times independently.

2. Computational procedures and data

Conventional MD simulations

Applying the program MODELLER (17), we constructed the F_O-domain from *M. abscessus* by using the BDQ bound F_O-domain from *M. smegmatis* (PDB-ID: 7JGC) (18) as a template. TBAJ-5307 was modelled by a least square fit procedure on BDQ. The protein-inhibitor complex was inserted into a 1-palmitoyl-2-oleoyl-sn-glycerol-3-phosphocholine (POPC) bilayer applying the membrane builder module (19) of the CHARMM graphical user interface (GUI) (20). Due to the presence of a lipid plug in the internal cavity of the *c*-ring (21), we also modelled a lipid plug into the internal cavity of the *c*-ring as described previously (22). A concentration of 0.15 M NaCl was added after solvation of the system and additional counter ions were inserted. To keep temperature and pressure constant at 300 K and 1 bar, we applied the velocity-rescale thermostat (23) and the Parrinello Rahman barostat (24). We simulated two sets of the system: 1) TBAJ-5307 and the key-glutamate (E62) were set charged and 2) TBAJ-5307 and E62 were set neutral. In both simulation setups we charged the E62 residue which is interacting with the essential arginine (R193) in subunit *a*. We used the CHARMM36m force field for proteins (25), the CHARMM36 force field for lipids (26), the CHARMM General force field (27) for TBAJ-5307 and the TIP3P water model (28). Simulations were carried out with GROMACS (version 2018), using a 2-fs integration time step. Electrostatic interactions were calculated with the Particle Mesh Ewald approach applying a real-space cut-off of 12 Å. Van-der-Waals interactions were switched after 8 Å with a cut-off of 12 Å. LINCS (29) was used to restrain bonds involving hydrogen atoms. All conventional MD simulations were carried out for 20 ns equilibration and 100 ns production time in triplicate.

Free energy calculations

We calculated the binding free energy of TBAJ-5307 using the thermodynamics integration (TI) approach. To prevent solvation of the key-glutamate (30) in its charged state and possible artefacts as a result of this solvation, we used the neutral state for E62 and TBAJ-5307 for these calculations. The coupling parameter λ was changed from 0 (drug fully present) to 1 (drug fully absent) using 27 windows. We simulated each window for 1 ns, discarding the first 100 ps as equilibration time. Subsequent analysis was performed with the BAR method (31). As TBAJ-5307 was restrained, an

entropic correction was applied. Three independent simulations, using the output structures of the three individual conventional MD runs as an input, were carried out.

3. Supplementary Figures

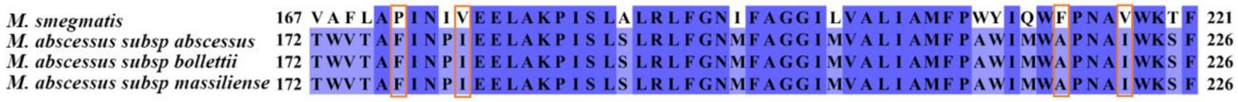

Supplementary Figure 1. (A) The sequence part of the *M. smegmatis* subunit *a* being in proximity to the BDQ-binding aligned with the three *M. abscessus* subspecies revealing significant differences in amino acid composition. The calculation of the percentage of identity was performed and presented in darker to lighter shades of *blue*, representing the most homologous to the least homologous. The proposed residues altering BDQ-binding in *M. smegmatis* and the *M. abscessus* complex strains are highlighted in a red box (32).

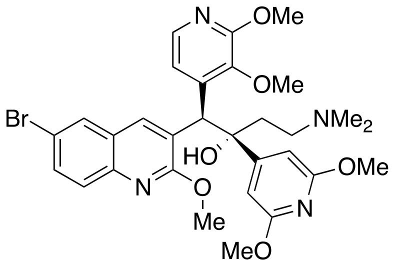

(±)-5307

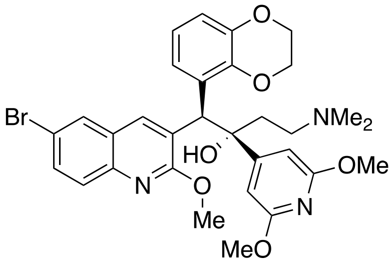

(±)-5316

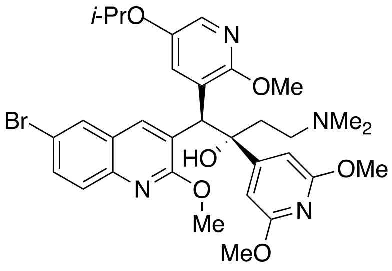

(±)-5366

Supplementary Figure 2. Structures of (±)-5307, (±)-5316, and (±)-5366.

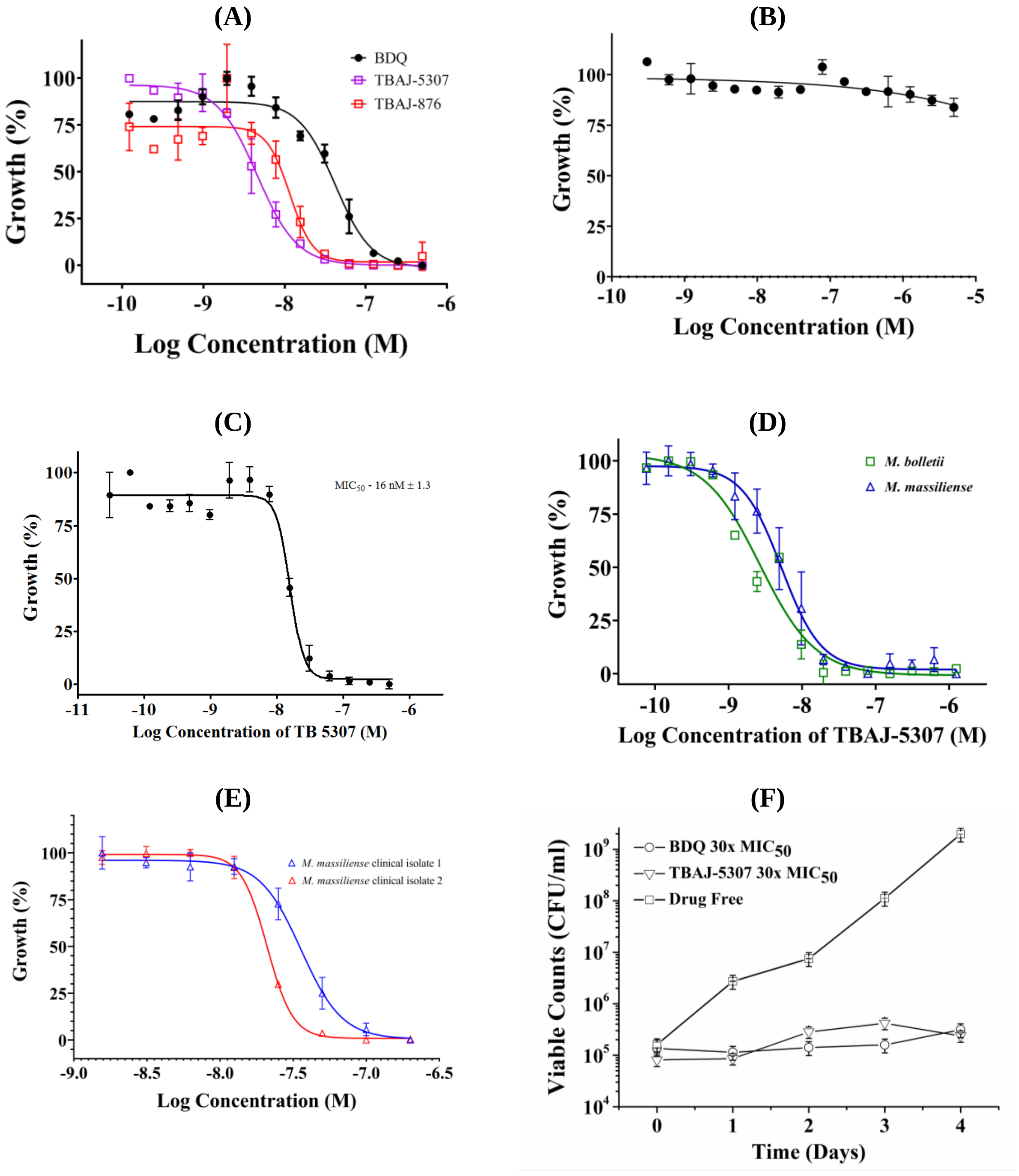
(A)
BDQ
TBAJ-5307
TBAJ-876
Growth (%)
Log Concentration (M)
(B)
Growth (%)
Log Concentration (M)
(C)
MIC50 - 16 nM ± 1.3
Growth (%)
Log Concentration of TB 5307 (M)
(D)
M. bolletii
M. massiliense
Growth (%)
Log Concentration of TBAJ-5307 (M)
(E)
M. massiliense clinical isolate 1
M. massiliense clinical isolate 2
Growth (%)
Log Concentration (M)
(F)
BDQ 30x MIC50
TBAJ-5307 30x MIC50
Drug Free
Viable Counts (CFU/ml)
Time (Days)

(G)

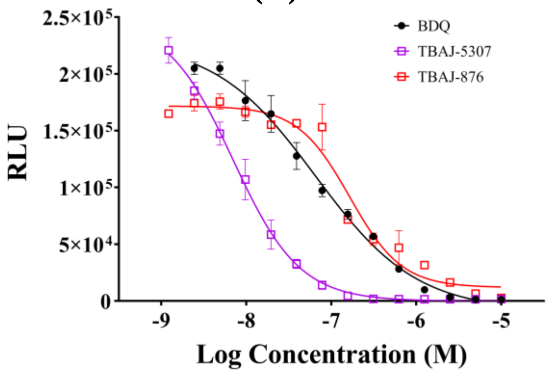

(H)

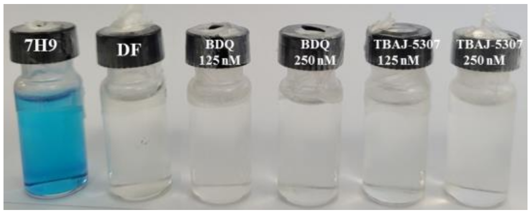

Supplementary Figure 3. (A) Dose-response curves of BDQ, TBAJ-876 and TBAJ-5307 against the rough morphotype strain of *M. abscessus* subsp. *abscessus.* Three independent experiments were carried out, each with three technical replicates. (B) In contrast to the high inhibitory potency of the enantiomer (-)-TBAJ5307 in (A), the enantiomer (+)-TBAJ5307, displayed no significant activity. TBAJ-5307 inhibits growth of the isolate *M. abscessus* Bamboo (C), the *M. abscessus* subclasses *M. bolletii* and *M. massiliense* (D) as well as *M. massiliense* clinical isolate 1 and *M. massiliense* clinical isolate 2 (E). Experiments were performed twice in triplicates. (F) Initial four days of untreated and TBAJ-5307 kill kinetics against *M. abscessus* subsp. a*bscessus*. The bacteria were grown in liquid culture (7H9) in the presence of the indicated concentrations of TBAJ-5307 up to 4 days. CFU was calculated by plating the culture on 7H10 agar plates. $P < 0.05$, statistical analysis was carried out for the experiment using ordinary one-way ANOVA test). Experiments were performed twice. (G) Intracellular ATP synthesis inhibition of rough morphotype strain of *M. abscessus* subsp. *abscessus* by TBAJ-5307. The ATP content of the cells was measured by adding bactiter glo (promega) to the cells. The total ATP content is directly proportional to relative luminescence units (RLU). Experiments were performed thrice in triplicates. (H) Oxygen consumption assay in *M. abscessus* using methylene blue as an oxygen sensor. TBAJ-5307 did also not affect oxygen consumption over a 72-h period. 7H9 media with dimethyl sulfoxide (DMSO) as compound solvent served as a blank/negative control. DF stands for drug free condition, including DMSO and *M. abscessus*, which served as a control for bacterial growth.

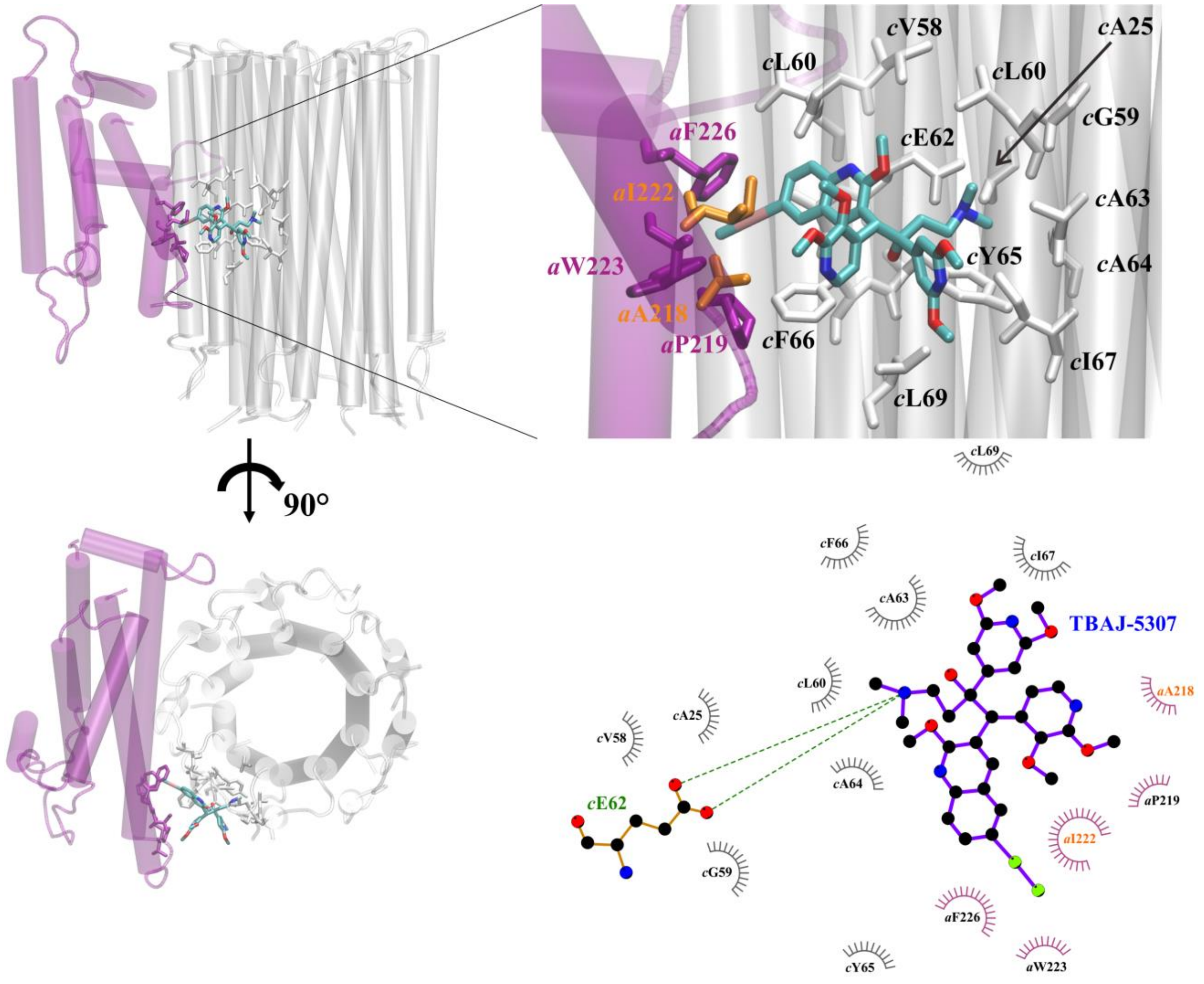

Supplementary Figure 4. Predicted binding of TBAJ-5307 to the *M. abscessus* F_O-leading site**.** Position of TBAJ-5307 bound to the F_O-domain in the side- (*top left*) and top view (*bottom left*), respectively. Zoom into the binding site (*top right*) and the LigPlot+ representation (33) (*bottom right*) are shown. Residues of the *c*-ring are displayed in grey and residues of subunit *a* are shown in purple (same residues as in *M. smegmatis*) and orange (different residues than in *M. smegmatis*). Figures representing molecular representations were produced with VMD (34).

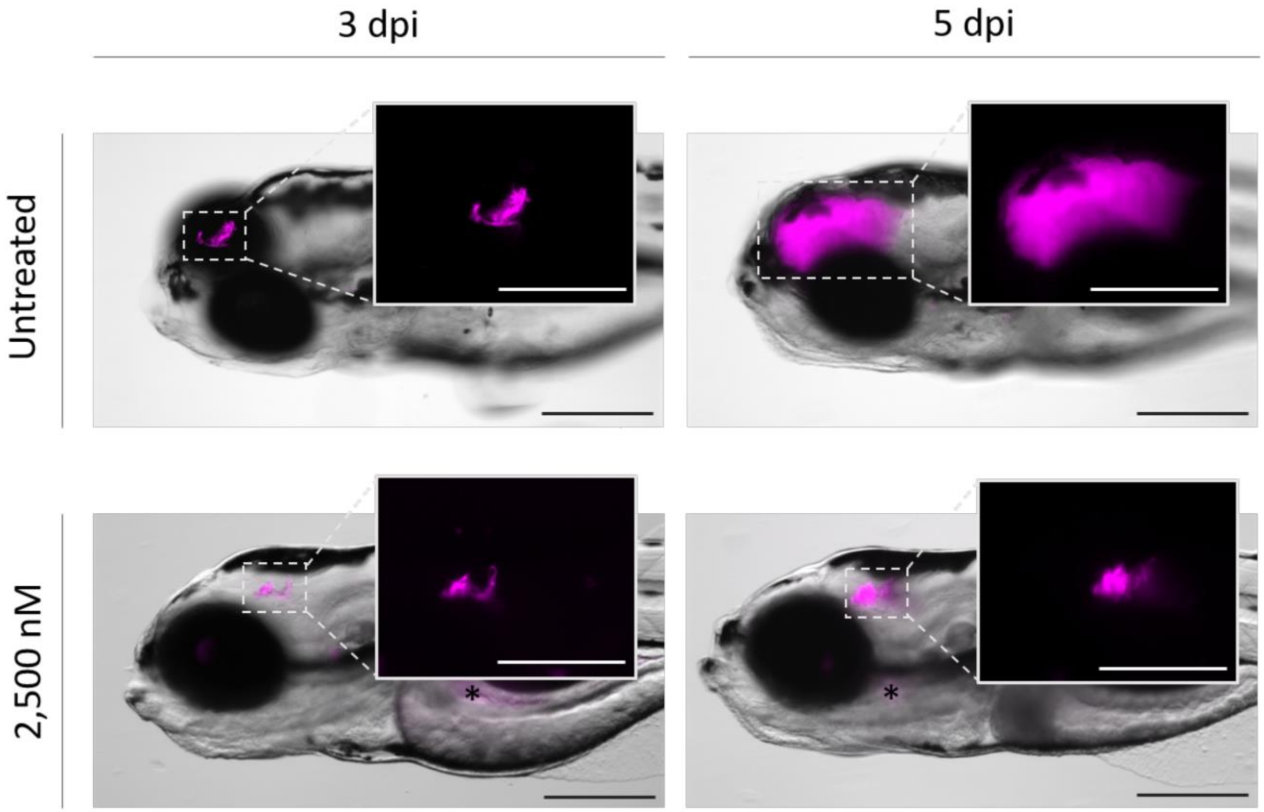

Supplementary Figure 5. Effect of TBAJ-5307 against *M. abcessus* CIP104536^{T} (R variant (35)) symptoms in an embryonic zebrafish infection model. Evolution of *M. abscessus* symptoms from cords (at 3 dpi) to abscesses (at 5 dpi) in embryos from the untreated group (*upper panels*) and from the treated group exposed to 2,500 nM TBAJ-5307 at 3 dpi (*left panels*) or 5 dpi (*right panels*). Scale bars in the main figures and insets represent 300 µm and 210 µm, respectively. * highlights autofluorescence.

(A)

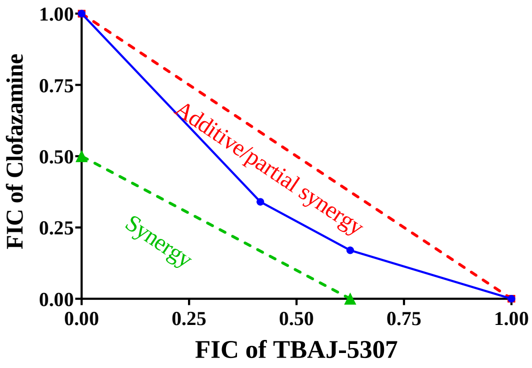

(B)

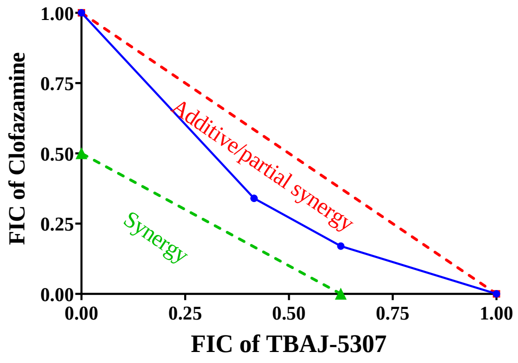

(C)

Drug A	Combination with drug B	MIC				FICI	Outcome
		Drug A alone	Drug A in combination	Drug B alone	Drug B in combination		
TBAJ-5307	Clofazamine	12 nM	5 nM	2.5 µM	0.85 µM	0.75	Additivity
TBAJ-5307	Rifabutin	12 nM	2.5 nM	1.5 µM	0.5 µM	0.77	Additivity

(D)

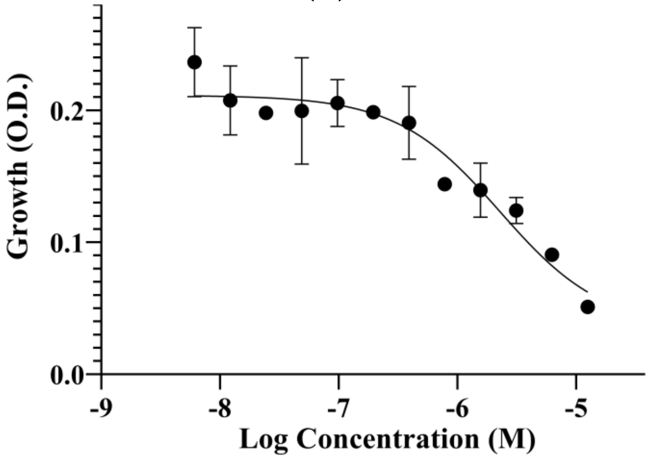

(E)

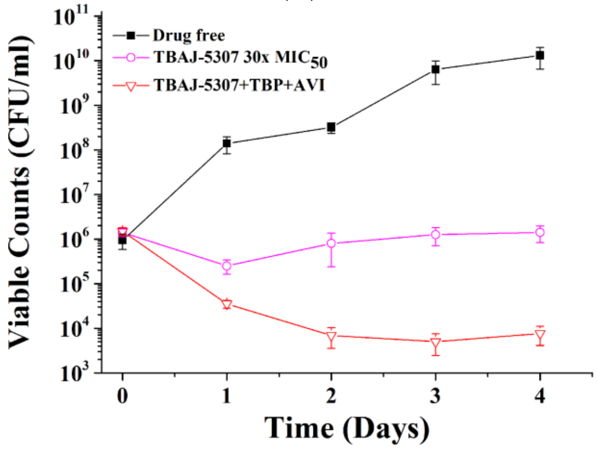

Supplementary Figure 6. Isobolograms of the combination of TBAJ-5307 and CFZ (A) as well as TBAJ-5307 and Rifabutin (RFB) (B) against *M. abscessus* subsp. *abscessus*. The green dash line indicates ideal isobole, where drugs act in synergy and independently. The red dash line indicates ideal isobole, where drugs act additively and in partial synergy. (C) The FICI was calculated as (MIC of drug A in combination/MIC of drug A alone) + (MIC of drug B in

combination/MIC of drug B alone). An FICI of ≤0.5 indicates synergy, a FICI of >0.5 to 4 indicates additivity (no interaction), and an FICI of >4 indicates antagonism (10). (D) Dose response growth inhibition of Tebipenam (TBP) with 4 µg/ml avibactam (AVI) against *M. abscessus* ATCC 19977. An MIC_{50} of 2 µM was observed in combination of the above two drugs. (E) Dose-response time-kill curves of TBAJ-5307, and TBAJ-5307+TBP+AVI against *M. abscessus* ATCC 19977. Cultures of *M. abscessus* ATCC 19977 were treated with 4x MIC (100 µM) TBP in combination with 4 mg/mL AVI for 4 days, and viability of the cultures was monitored by CFU determination.

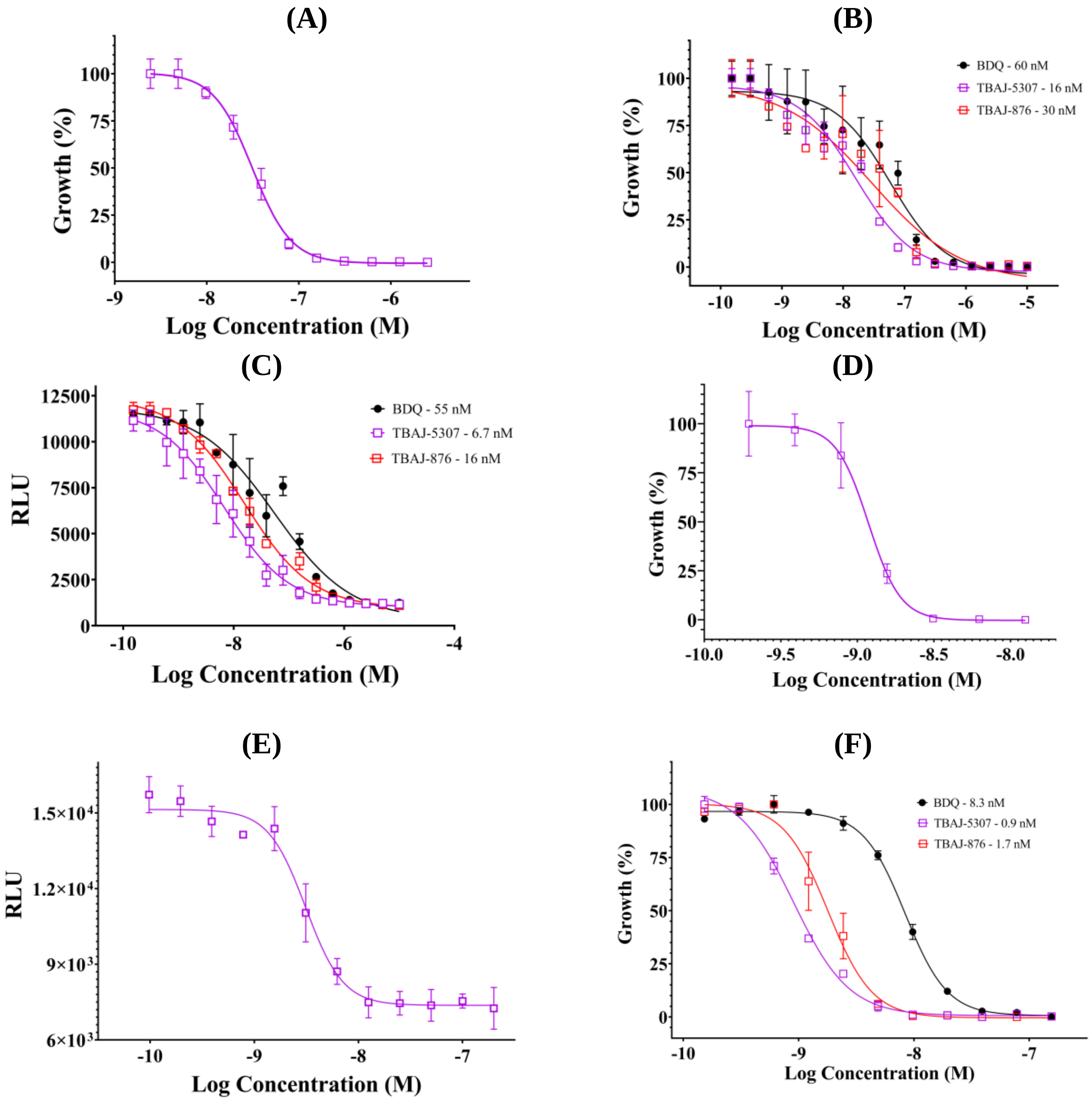

Supplementary Figure 7 (A) TBAJ-5307 inhibits growth of the clinical isolate *M. avium* 11. (B) Growth inhibition dose response curve of *M. intracellulare* by TBAJ-5307 in comparison to BDQ and TBAJ-876. Three independent experiments were carried out, each with three technical replicates. (C) TBAJ-5307 inhibits oxidative phosphorylation in a whole cell ATP synthesis assay better compared to BDQ and TBAJ-876. The total ATP content is directly proportional to relative luminescence units (RLU). The experiments have been performed in triplicates. (D) *M. mucogenicum* growth- and ATP synthesis (E) inhibition by TBAJ-5307. (F) Dose response curve of *M. fortuitum* growth inhibition by TBAJ-5307 in comparison to BDQ and TBAJ-876. Three independent experiments were carried out, each with three technical replicates.

4. Supplementary Tables

Supplementary Table 1: Mean minimum distance of the *Mab* F_O-domain residues to TBAJ-5307 during simulations. Distances are shown for TBAJ-5307 bound to each site (leading, lagging and *c*-ring only) when drug and key-glutamate are charged or protonated. Distances are reported in Å. For each protonation state, data were calculated over a total of three replicates of 100 ns.

	**TBAJ-5307 (deprotonated)**			**TBAJ-5307 (protonated)**		
	***c*-ring**	**Lagging**	**Leading**	***c*-ring**	**Lagging**	**Leading**
***c*A25**	5.1 ± 0.6	5.2 ± 0.6	5.0 ± 0.7	5.5 ± 0.8	5.2 ± 0.6	5.4 ± 0.6
***c*V58**	4.9 ± 0.5	4.9 ± 0.5	5.1 ± 0.6	5.6 ± 0.5	5.7 ± 0.4	5.4 ± 0.5
***c*G59**	4.1 ± 0.3	4.1 ± 0.3	4.3 ± 0.4	4.4 ± 0.5	4.4 ± 0.3	4.4 ± 0.3
***c*L60**	4.0 ± 0.3	3.9 ± 0.3	4.0 ± 0.3	4.3 ± 0.5	4.4 ± 0.4	4.2 ± 0.4
***c*E62**	3.5 ± 0.1	3.5 ± 0.1	3.5 ± 0.1	3.6 ± 0.2	3.6 ± 0.2	3.6 ± 0.2
***c*E62:Oεx -drug:N**	2.7 ± 0.1	2.7 ± 0.1	2.8 ± 0.4	2.9 ± 0.4	2.8 ± 0.1	2.8 ± 0.5
***c*A63**	3.6 ± 0.2	3.6 ± 0.2	3.6 ± 0.2	3.6 ± 0.3	3.6 ± 0.2	3.6 ± 0.2
***c*A64**	3.8 ± 0.2	3.8 ± 0.2	4.0 ± 0.3	4.0 ± 0.6	3.8 ± 0.2	3.9 ± 0.3
***c*Y65**	3.8 ± 0.2	3.8 ± 0.2	3.8 ± 0.2	3.8 ± 0.5	3.7 ± 0.2	3.8 ± 0.2
***c*F66**	3.6 ± 0.2	3.6 ± 0.2	3.6 ± 0.2	3.6 ± 0.2	3.6 ± 0.2	3.6 ± 0.2
***c*I67**	3.6 ± 0.2	3.6 ± 0.2	3.6 ± 0.2	3.6 ± 0.3	3.7 ± 0.2	3.6 ± 0.2
***c*L69**	3.7 ± 0.6	3.6 ± 0.4	3.8 ± 0.6	4.0 ± 1.0	3.6 ± 0.4	4.5 ± 1.0
***a*V174**	N/A	5.1 ± 0.9	N/A	N/A	5.5 ± 1.1	N/A
***a*F177**	N/A	3.7 ± 0.4	N/A	N/A	3.7 ± 0.4	N/A
***a*I178**	N/A	3.6 ± 0.3	N/A	N/A	3.7 ± 0.3	N/A
***a*I181**	N/A	4.4 ± 0.9	N/A	N/A	3.9 ± 0.3	N/A
***a*A218**	N/A	N/A	4.6 ± 1.0	N/A	N/A	4.0 ± 0.4
***a*P219**	N/A	N/A	4.8 ± 0.9	N/A	N/A	4.1 ± 0.5
***a*I222**	N/A	N/A	3.7 ± 0.2	N/A	N/A	3.7 ± 0.2
***a*W223**	N/A	N/A	3.0 ± 1.5	N/A	N/A	2.9 ± 1.2
***a*F226**	N/A	N/A	2.8 ± 0.5	N/A	N/A	2.9 ± 0.6

5. References

1. M. Yee, D. Klinzing, J. R. Wei, M. Gengenbacher, E. J. Rubin, T. Dick, *Gen. Announc.* **2017**, *5(20)*.
2. M. Yee, D. Klinzing, J. R. Wei, M. Gengenbacher, E. J. Rubin, J. Y. Chien, P. R. Hsueh, T. Dick, *Gen. Announc.* **2017**, *5(32)*.
3. H.-Y. Kim, B. J. Kim, Y. Kook, Y.-J. Yun, J. H. Shin, B.-J. Kim, Y.-H. Kook, *Microbiol. Immunol.* **2010**, *54*, 347-353.
4. W. Moreira, D. B. Aziz, T. Dick, *Front. Microbiol.* **2016**, *7*.
5. P. Ragunathan, T. Dick, G. Grüber, *Antimicrob. Agents Chemother.* **2022**, *66*, e0001822.
6. C. A. Motulsky H, *Fitting models to biological data using linear and nonlinear regression: a practical guide to curve fitting. GraphPad software, Inc, San Diego, CA. www.graphpad.com.* , **2003**.
7. A. Hotra, P. Ragunathan, P. S. Ng, P. Seankongsuk, A. Harikishore, J. P. Sarathy, W.-G. Saw, U. Lakshmanan, P. Sae-Lao, N. P. Kalia, J. Shin, R. Kalyanasundaram, S. Anbarasu, K. Parthasarathy, C. N. Pradeep, H. Makhija, P. Dröge, A. Poulsen, J. H. L. Tan, K. Pethe, T. Dick, R. W. Bates, G. Grüber, *Ang. Chem. Intern. Edi.* **2020**, *59*, 13295-13304.
8. M. H. Hsieh, C. M. Yu, V. L. Yu, J. W. Chow, *Diagn. Microbiol. Inf. Dis.* **1993**, *16*, 343-349
9. A. Kaushik, N. Makkar, P. Pandey, N. Parrish, U. Singh, G. Lamichhane, *Antimicrob. Agents Chemother.* **2015**, *59*, 6561-6567.
10. F. C. Odds, *J. Antimicrob. Chemoth.* **2003**, *52*, 1.
11. D. N. Schleheck, N. Barraud, J. Klebensberger, J. S. Webb, D. McDougald, S. A. Rice, S. Kjelleberg, *PLoS One* **2009,** *4(5)*: e5513.

12 W. H. Poh, N. Barraud, S. Guglielmo, L. Lazzarato, B. Rolando, R. Fruttero, S. A. Rice *ACS Chemical Biology* **2017,** *12(8)*: 2097-2106.

13. A. Bernut, J. L. Herrmann, K. Kissa, J. F. Dubremetz, J. L. Gaillard, G. Lutfalla, L. Kremer, *Proc. Natl. Acad. Sci. USA* **2014**, *111*, E943-952.
14. R. L. Lamason, M. A. Mohideen, J. R. Mest, A. C. Wong, H. L. Norton, M. C. Aros, M. J. Jurynec, X. Mao, V. R. Humphreville, J. E. Humbert, S. Sinha, J. L. Moore, P. Jagadeeswaran, W. Zhao, G. Ning, I. Makalowska, P. M. McKeigue, D. O'Donnell, R. Kittles, E. J. Parra, N. J. Mangini, D. J. Grunwald, M. D. Shriver, V. A. Canfield, K. C. Cheng, *Science (New York, N.Y.)* **2005**, *310*, 1782-1786.
15. A. Bernut, C. Dupont, A. Sahuquet, J. L. Herrmann, G. Lutfalla, L. Kremer, *J. Vis. Exp.* **2015**.
16. A. Bernut, V. Le Moigne, T. Lesne, G. Lutfalla, J. L. Herrmann, L. Kremer, *Antimicrob. Agents Chemother.* **2014**, *58*, 4054-4063.
17. A. Fiser, A. Sali, *Meth. Enzymol.* **2003**, *374*, 461-491.
18. H. Guo, G. M. Courbon, S. A. Bueler, J. Mai, J. Liu, J. L. Rubinstein, *Nature* **2021**, *589*, 143-147.
19. E. L. Wu, X. Cheng, S. Jo, H. Rui, K. C. Song, E. M. Dávila-Contreras, Y. Qi, J. Lee, V. Monje-Galvan, R. M. Venable, J. B. Klauda, W. Im, *J. Comp. Chem.* **2014**, *35*, 1997-2004.
20. S. Jo, T. Kim, V. G. Iyer, W. Im, *J. Comp. Chem.* **2008**, *29*, 1859-1865.
21. T. Meier, U. Matthey, F. Henzen, P. Dimroth, D. J. Müller, *FEBS Lett.* **2001**, *505*, 353-356.

22. A. Krah, J. K. Marzinek, P. J. Bond, *J. Phys. Chem. B* **2020**, *124*, 7176-7183.
23. G. Bussi, D. Donadio, M. Parrinello, *J. Chem. Phys.* **2007**, *126*, 014101.
24. M. Parrinello, A. Rahman, *J. Appl. Phys.* **1981**, *52*, 7182-7190.
25. J. Huang, S. Rauscher, G. Nawrocki, T. Ran, M. Feig, B. L. de Groot, H. Grubmüller, A. D. MacKerell, Jr., *Nat. Meth.* **2017**, *14*, 71-73.
26. J. B. Klauda, R. M. Venable, J. A. Freites, J. W. O'Connor, D. J. Tobias, C. Mondragon-Ramirez, I. Vorobyov, A. D. MacKerell, Jr., R. W. Pastor, *J. Phys. Chem. B* **2010**, *114*, 7830-7843.
27. K. Vanommeslaeghe, E. Hatcher, C. Acharya, S. Kundu, S. Zhong, J. Shim, E. Darian, O. Guvench, P. Lopes, I. Vorobyov, A. D. Mackerell, Jr., *J. Comp. Chem.* **2010**, *31*, 671-690.
28. W. L. Jorgensen, J. Chandrasekhar, J. D. Madura, R. W. Impey, M. L. Klein, *J. Chem. Phys.* **1983**, *79*, 926-935.
29. B. Hess, Bekker, H., Berendsen, H. and Fraaije, J., *J. Comp. Chem.* **1997**, *18*, 1463-1472.
30. a) H. Gohlke, D. Schlieper, G. Groth, *J. Biol. Chem.* **2012**, *287*, 36536-36543; b) A. Krah, D. Pogoryelov, T. Meier, J. D. Faraldo-Gómez, *J. Mol. Biol.* **2010**, *395*, 20-27.
31. C. H. Bennett, *J. Comp. Phys.* **1976**, *22*, 245-268.
32 A. Krah, P. Ragunathan, P. J., Bond, G. Grüber, *Biochem. Biophys. Res. Comm.* **2023,** 690:149249. doi: 10.1016/j.bbrc.2023.149249
33. R. A. Laskowski, M. B. Swindells, *J. Chem. Inf. Model.* **2011**, *51*, 2778-2786.
34. W. Humphrey, A. Dalke, K. Schulten, *J. Mol. Graph.* **1996**, *14*, 33-38, 27-38.
35. F. Ripoll, S. Pasek, C. Schenowitz, C. Dossat, V. Barbe, M. Rottman, E. Macheras, B. Heym, J. L. Herrmann, M. Daffé, R. Brosch, J. L. Risler, J. L. Gaillard, *PLoS One.* **2009,** *4(6)*:e5660.
